# Ethyl ester/acyl hydrazide-based aromatic sulfonamides: facile synthesis, structural characterization, electrochemical measurements and theoretical studies as effective corrosion inhibitors for mild steel in 1.0 M HCl†

**DOI:** 10.1039/d2ra05939h

**Published:** 2022-12-20

**Authors:** Mahmoud A. Bedair, Ahmed M. Abuelela, Mubark Alshareef, Medhat Owda, Essam M. Eliwa

**Affiliations:** Department of Chemistry, Faculty of Science (Men's Campus), Al-Azhar University Nasr City 11884 Cairo Egypt m_bedier@azhar.edu.eg m_bedier@yahoo.com ahmed.abuelela@azhar.edu.eg; College of Science and Arts, University of Bisha P.O. Box 101 Al-Namas 61977 Saudi Arabia mbedair@ub.edu.sa; Department of Chemistry, Faculty of Applied Science, Umm Al Qura University Makkah 24230 Saudi Arabia mmshreef@uqu.edu.sa

## Abstract

In this research paper, aromatic sulfonamide-derived ethyl ester (*p*-TSAE) and its acyl hydrazide (*p*-TSAH) were directly synthesized, characterized, and employed for the first time as prospective anticorrosive agents to protect mild steel in 1.0 M HCl conditions. The corrosion efficiency was probed by electrochemical methods including polarization, impedance, and frequency modulation measurements. Optimal efficiencies of 94% and 92% were detected for the hydrazide and ester, respectively, revealing excellent corrosion inhibition. Moreover, both the hydrazide and ester molecules combat the cathodic and anodic reactions correspondingly in a mixed-type manner. The electron transfer (ET) at the inhibitor/metal interface was evaluated using DFT at the B3LYP/6-31g(d,p) level. Natural bond orbital analysis (NBO) and frontier molecular orbital analysis (FMO) calculations showed superior capabilities of the synthesized inhibitors to easily reallocate charge into the metal surface. However, the hydrazide molecules showed slightly better inhibition efficiency than the ester due to the strong interaction between the lone pairs of the nitrogen atoms and the d-orbitals of the metal. The chemical hardness of the hydrazide and ester are 2.507 and 2.511 eV, respectively, in good accordance with the recorded electrochemical inhibition efficiencies for both molecules. Good and straightforward correlations between the experiments and calculations are obtained.

## Abstract 2

Two new corrosion inhibitors were synthesized and applied to steel in 1.0 M HCl. The corrosion resistance was probed by electrochemical and theoretical methods. The study reveals significant intersections between electrochemical aspects and theory.

## Graphical abstract

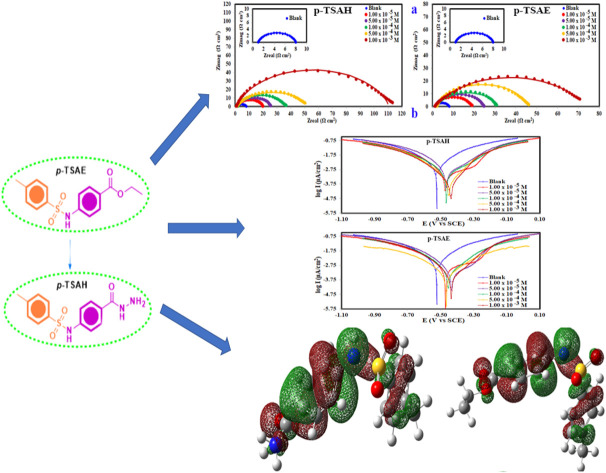

## 1. Introduction

Organic molecules are widely used as effective corrosion inhibitors for mild steel in aqueous media as they are rich in adsorption centers, *i.e.* hetero atoms (O, N and S), and a small amount of the inhibitor can provide a high inhibition efficiency, which makes it cost effective.^1–3^ The rate of both cathodic and anodic corrosion reactions at the metal/solution interface can be suppressed to a high extent by the mutual interactions between the metal surface and the organic film, which are governed by an adsorption mechanism.^4,5^ While the rate of corrosion can be probed by electrochemical methods such as electrochemical impedance spectroscopy, electrochemical frequency modulation and potentiodynamic polarization, the metal–inhibitor interactions can be probed by theoretical calculations.^6,7^

Aromatic/heterocyclic sulfonamides are a broad collection of privileged organic sulfur motifs that have received a lot of interest in the pharmaceutical industry as drugs and potential therapeutic candidates.^8^ In 2020, Mondal and Malakar documented in their review article the advanced protocols for the chemical synthesis of sulfonamides and their important synthetic and biological implementations.^9^ More recently, Iakovenko *et al.* (2022) described a novel approach for the synthesis of *N*-alkyl heteroaryl sulfonamides in good yields *via* the direct oxidative coupling of readily available heterocyclic thiols and primary amines under mild conditions.^10^ This landmark work has been extended to transform the prior products to the corresponding *N*,*N*-dialkyl heteroaryl sulfonamides through a microwave-assisted Fukuyama–Mitsunobu alkylation reaction.^10^ In 2021, Mkrtchyan and Iaroshenko reported a successful mechanochemical strategy to construct diverse aromatic sulfonamides by the Pd-mediated one-pot coupling transformation of K_2_S_2_O_5_ (a source of SO_2_) and amines with aryl bromides or aromatic carboxylic acids as arylation reagents.^11^

Abd El-Lateef's (2020) study showed that a new salicylidene isatin hydrazone sodium sulfonate inhibits the corrosion of carbon steel (CS) in 1.0 M HCl by up to 87.8%, and when merged with nickel (Ni^2+^) cations, the inhibition ability was increased up to 99.2%.^12^ In 2021, Verma and his colleagues^13^ reported the synthesis and characterization of three *N*-hydroxybenzothioamide analogues that exhibited inhibition efficiencies of over 90% at concentrations of 300 ppm. In March 2021, Abd El-Lateef *et al.*'s published article^14^ described the construction of a new hydrazide–hydrazone and its metal complexes with Zn(ii) and ZrO(ii), derived from nicotinic acid hydrazide and salicylaldehyde-5-sodium sulfonate salt. Chelate-based ZrO showed the best anti-corrosive potency with an efficacy reaching 97.4% at a dose of 5 × 10^−4^ mol L^−1^. In fact, several hydrazides and hydrazones have attracted a high level of attention in corrosion studies recently, as shown in Table 1.^15–22^ This table shows a comparison of recent reports and currently investigated compounds as corrosion inhibitors for mild steel in 1.0 M HCl, of which our investigated compounds show significant and promising efficiencies compared to the other published compounds.

**Table 1:** Comparison of our outcomes with previously reported studies on the employment of hydrazides as anti-corrosive agents

Inhibitor	Conc.	Metal substrate	Corrosive media	Temp.	Inhibition efficiency^a^ (%)	Ref.
Acyl hydrazide–hydrazones containing acetophenone moiety	300 mg L^−1^	Mild steel	1.0 M HCl	308 K	85.58–85.73	15
Acyl hydrazide–hydrazones containing diphenylamine moiety	5 × 10^−3^ mol L^−1^	Mild steel	1.0 M HCl	303 K	84.0–95.0	16
Acyl hydrazide–hydrazones containing pyridinium salt	1 × 10^−3^ mol L^−1^	Mild steel	1.0 M HCl	298 K	88.8–92.3	17
Acyl hydrazide–hydrazones containing pyrazole moiety	1 × 10^−3^ mol L^−1^	Mild steel	1.0 M HCl	298 K	75.7–93.0	18
Acyl hydrazide–hydrazones containing naphthalene moiety	5 × 10^−3^ mol L^−1^	Mild steel	1.0 M HCl	303 K	90.0–95.0	19
Acyl hydrazides containing indole moiety	1 × 10^−3^ mol L^−1^	Mild steel	0.5 M HCl	303 K	74.3–97.0	20
Diacyl hydrazide–hydrazones containing ethanoanthracene moiety	1 × 10^−4^ mol L^−1^	N80 steel	3.5% NaCl	25 °C	91.6–92.2	21
Diacyl hydrazide–hydrazone–derived cationic gemini surfactant	1 × 10^−4^ mol L^−1^	X-65 steel	1.0 M HCl	25 °C	91.44	22
Acyl hydrazide containing sulfonamide moiety (*p*-TSAH)	1.00 × 10^−3^ M	Mild steel	1.0 M HCl	25 °C	93.73	This study

a Inhibition efficiencies (%) are tabulated according to the electrochemical impedance spectroscopy (EIS) measurements.

A combination of the hydrazide functional group and sulfur-containing compounds such as sulfonamides for application as corrosion inhibitors is rare in the literature, indicating a gap that needs to be filled. According to the best of our information, there is one research article, published in 2020 by Laggoun and co-authors, who described the protection impact of *p*-toluenesulfonyl hydrazide (*p*-TSH) on the copper surface in a 0.5 M HCl medium.^23^ In a promising result, *p*-TSH successfully blocked the corrosion sites on the copper surface with an approximate inhibition efficiency of 90% at 5 mM. Consequently, this finding encouraged us to synthesize ethyl 4-(4-methylphenylsulfonamido)benzoate (*p*-TSAE, 8) and 4-(4-methylphenyl-sulfonamido)benzohydrazide (*p*-TSAH, 9), and utilize them for the first time ever as potential anticorrosive agents to protect mild steel under acidic conditions due to 1.0 M HCl. We used DFT calculations with the B3LYP method and 6-31g(d,p) basis set to obtain molecular reactivity parameters on both the molecular and atomic levels. New and promising findings were obtained relating the molecular aspects of the synthesized inhibitors to mutual inhibitor–metal interactions.

## 2. Experimental and computational details

### 2.1 Materials and instrumental techniques

For the chemical synthesis, starting materials and reagents were obtained from commercial sources, particularly Acros Organics™ (Geel, Belgium), and used without further purification unless otherwise indicated. Solvents were obtained from Thermo Fisher Scientific™ and employed without additional purification. All reactions were carried out in oven-dried glassware. The purity of the synthesized compounds was investigated by TLC and performed on Merck precoated silica gel 60 F_254_ aluminum sheets with a solvent mixture of DCM–MeOH (99–1) as the eluent system. Spots were visualized under UV illumination at 254 nm. Chemical synthesis procedures were not optimized. Compound names are derived from ChemOffice Suite 2021 v21.0.0.28 and are not necessarily identical to those according to IUPAC nomenclature. Schemes were produced by the same version of ChemOffice. IR and MS data were plotted by OriginPro 2021. NMR spectra were delineated *via* MestReNova v12.0.2.

Melting points were determined on a SMP50 Digital APP (Bibby Scientific, Staffordshire, UK) 120/230 V, in open capillaries and are uncorrected. The mid-IR spectra (KBr, *ν*/cm^−1^) were recorded on a CARY 630 FT-IR spectrophotometer (Agilent, Santa Clara, CA, USA). NMR spectra (^1^H NMR and ^13^C NMR) were recorded on a Bruker EX-500 MHz (^1^H: 500 MHz, ^13^C: 125 MHz) spectrometer (Bruker, USA) at room temperature. Tetramethylsilane (TMS) was used for internal calibration (^1^H NMR and ^13^C NMR: 0.00 ppm). Chemical shifts were reported in parts per million (ppm) on the *δ* scale and relative to residual solvent peaks (DMSO-*d*_6_: ^1^H: 2.50 ppm, ^13^C: 39.5 ppm). Coupling constants (*J*) are reported in Hz with the following abbreviations used to indicate splitting: s = singlet, d = doublet, t = triplet, q = quartet, m = multiplet, br = broad signal. Electron ionization mass spectra (EI-MS) were obtained using a GC MS-QP 2010 plus mass spectrometer (Shimadzu, Kyoto, Japan) at an electron voltage of 70 eV, with a time of 10 min, scan speed of 1000, and *m*/*z* range of 50–500.

### 2.2 Synthesis and characterization measurements

#### 2.2.1 Chemical synthesis of ethyl 4-(4-methylphenylsulfonamido)benzoate (*p*-TSAE, 8, CAS: 739-33-3)^24,25^

**Scheme 1:**
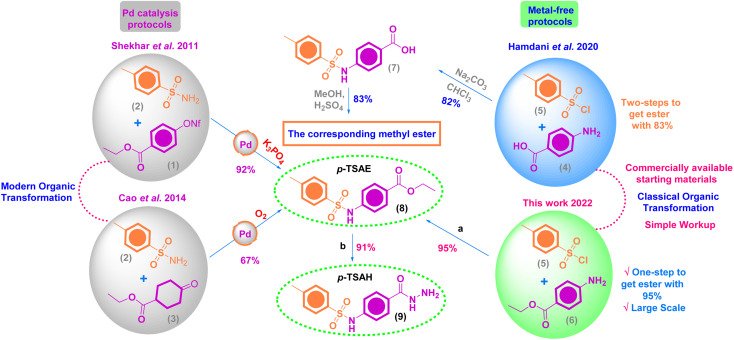
Previous organic transformations and our approach for the chemical synthesis of 8 and 9. Reagents and conditions: (a) 1 (16.5 g, 0.1 mol, 1.0 equiv.), 2 (19.1 g, 0.1 mol, 1.0 equiv.), DCM, TEA (10.1 g, 0.1 mol, 14.4 mL, 1.0 equiv.), stirring, 8 h, 95% yield; (b) 8 (16 g, 0.05 mol, 1.0 equiv.), NH_2_NH_2_·H_2_O 100% (10 mL, 0.2 mol, 4.0 equiv.), pyridine (3.95 g, 4.0 mL, 0.05 mol, 1.0 equiv.), EtOH, reflux, 10 h, 91% yield.


For the large-scale synthesis under a nitrogen atmosphere, into a 500 mL, two-necked, oven dried flat-bottomed flask that was equipped with a magnetic stir bar, ethyl 4-aminobenzoate (6, benzocaine, CAS: 94-09-7, 16.5 g, 0.1 mol, 1.0 equiv.) was dissolved in dichloromethane (DCM, 50 mL) containing triethylamine (TEA, 10.1 g, 0.1 mol, 14.4 mL, 1.0 equiv.) (Scheme 1). A solution of 4-toluenesulfonyl chloride (TsCl, 5, CAS: 98-59-9, 19.1 g, 0.1 mol, 1.0 equiv.) in DCM (50 mL) was added portionwise over 30 min and the reaction mixture was stirred at room temperature for 8 h. The precipitated solid was isolated *via* vacuum filtration and washed with DCM (3 × 20 mL), then air dried. The obtained ester 8 was employed in the next step without additional purification.

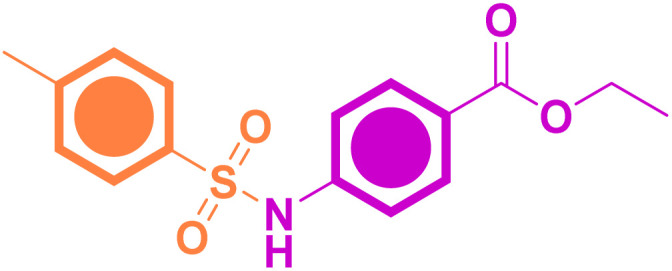
Off-white amorphous solid; yield: 95%; mp 205 °C (lit. mp 202–206 °C (ref. 24)); mid FT-IR (KBr, *ν*/cm^−1^) 3216 (*N–H stretching*), 3062, 2985 (*aromatic C–H stretching*), 2942, 2904 (*aliphatic C–H stretching*), 1693 (*ester C=O stretching*), 1604 (*C=C stretching*), 1511 (*N–H bending*), 1477 (*aliphatic C–H bending*), 1338 (*asym. O=S=O stretching*), 1288 (*aromatic amine C–N stretching*), 1234 (*aromatic ester C–O stretching*), 1158 (*sym. O=S=O stretching*); ^1^H NMR (500 MHz, DMSO-*d*_6_, ppm) *δ* 10.81 (s, 1H, N***H***), 7.80–7.76 (m, 2H, ArC***H***), 7.71–7.67 (m, 2H, ArC***H***), 7.28 (d, *J* = 8.0 Hz, 2H, ArC***H***), 7.22–7.19 (m, 2H, ArC***H***), 4.17 (q, *J* = 7.1 Hz, 2H, ester C***H***_***2***_), 2.24 (s, 3H, ArC***H***_***3***_), 1.19 (t, *J* = 7.1 Hz, 3H, ester C***H***_***3***_). ^13^C NMR (125 MHz, DMSO, ppm) *δ* 165.66 (ester ***C***=O), 144.11 (*quat*-***C***-CH_3_), 142.99 (*quat*-***C***-NH), 137.02 (*quat*-***C***-SO_2_), 131.05 (Ar***C***H), 130.25 (Ar***C***H), 127.30 (Ar***C***H), 125.21 (*quat*-***C***-COOEt), 118.63 (Ar***C***H), 118.57 (Ar***C***H), 60.92 (ester ***C***H_2_), 21.36 (Ar***C***H_3_), 14.58 (ester ***C***H_3_); EI-MS for C_16_H_17_NO_4_S [M]^+^*m*/*z* (%): 319 (30), 274, 226, 182, 155, 92, 91 (100), 65.

#### 2.2.2 Chemical synthesis of 4-(4-methylphenylsulfonamido)benzohydrazide (*p*-TSAH, 9)^26^

An oven-dried 500 mL, one-necked, flat-bottomed flask, equipped with a magnetic stirring bar, was charged with carboxylate ester 8 (16 g, 0.05 mol, 1.0 equiv.) in EtOH (150 mL) containing pyridine (3.95 g, 4.0 mL, 0.05 mol, 1.0 equiv.). Next, the reaction mixture was heated at 60 °C for 10 min to activate the ester group. After that, hydrazine hydrate 100% (10 mL, 0.2 mol, 4.0 equiv.) was added and the reaction mixture was heated under reflux conditions in an oil bath for 10 h. A colorless solid was formed while hot and the reaction solution was then cooled to room temperature. The precipitated solid was collected by vacuum filtration and washed with hot EtOH (3 × 20 mL), then air dried.


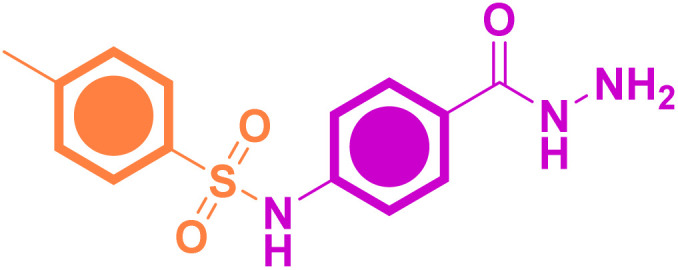
Colorless amorphous solid; yield: 91%; mp 272–274 °C (lit. mp 270–272 °C (ref. 26)); mid FT-IR (KBr, *ν*/cm^−1^) 3321, 3279 (*NH*_*2*_*stretching*), 3146 (*N–H stretching*), 3048, 2940 (*aromatic C–H stretching*), 2875 (*aliphatic C–H stretching*), 1649 (*hydrazide C=O stretching*), 1607 (*C=C stretching*), 1579, 1536 (*N–H bending*), 1476 (*aliphatic C–H bending*), 1335 (*asym. O=S=O stretching*), 1292 (*aromatic amine C–N stretching*), 1152 (*sym. O=S=O stretching*); ^1^H NMR (500 MHz, DMSO-*d*_6_, ppm) *δ* 10.51 (s, 1H, N***H***), 9.63–9.59 (m, 1H, N***H***), 7.67 (dd, *J* = 8.2, 2.6 Hz, 4H, ArC***H***), 7.31 (d, *J* = 8.0 Hz, 2H, ArC***H***), 7.12 (d, *J* = 8.5 Hz, 2H, ArC***H***), 4.45–4.38 (m, 2H, N***H***_***2***_), 2.27 (s, 3H, ArC***H***_***3***_); ^13^C NMR (126 MHz, DMSO, ppm) *δ* 165.93 (hydrazide ***C***=O), 144.09 (*quat*-***C***-CH_3_), 140.99 (*quat*-***C***-NH), 137.01 (*quat*-***C***-SO_2_), 130.35 (*quat*-***C***-CONHNH_2_), 128.85 (Ar***C***H), 128.75 (Ar***C***H), 127.28 (Ar***C***H), 118.82 (Ar***C***H), 21.48 (Ar***C***H_3_); EI-MS for C_14_H_15_N_3_O_3_S [M + 2]^+^*m*/*z* (%): 307 (7), 295, 241, 196, 162, 108, 92, 91 (100), 63.

### 2.3 Electrochemical measurements

A three-electrode cell containing a fresh 100 ml aerated solution of 1.00 M HCl with/without different concentrations of the synthesized inhibitors was first left for 1 h to obtain a steady state open circuit potential. After the solution had reached the open circuit potential (*E*_OCP_), three consecutive electrochemical techniques, electrochemical frequency modulation (EFM), electrochemical impedance spectroscopy (EIS) and potentiodynamic polarization (PDP) were conducted at a temperature of 303.15 K. The first cell electrode was a mild steel working electrode (WE), with a total exposed surface area of 1 cm^2^ and elemental composition of 97.929% Fe, 0.330% C, 0.030% Mo, 0.836% Mn, 0.043% Al, 0.389% Si, 0.157% Cr, 0.033% S, 0.075% Ni, 0.014% Co, 0.149% Cu, and 0.015% W. The second electrode was a graphite counter electrode (CE), and the third was the saturated calomel electrode (SCE) as a reference electrode. The investigated inhibitor concentrations were 1.00 × 10^−5^, 5.00 × 10^−5^, 1.00 × 10^−4^, 5.00 × 10^−4^ and 1.00 × 10^−3^ M. EFM was achieved by applying 2 Hz and 5 Hz frequencies, with a base frequency of 0.1 Hz, and a perturbation signal of 10 mV, for 16 cycles of repeated sinusoidal waveform. The highest frequency should be at least twice the lower one, and slow enough to eliminate the double layer charging contribution to the current response. EIS measurements were carried out at the OCP in a frequency range from 1.0 × 10^5^ to 1.0 × 10^−1^ Hz using 10 mV as the AC sinusoidal perturbation. PDP tests were carried out from −500 mV to 500 mV against the OCP with a sweeping rate of 0.5 mV s^−1^. Prior to each experiment, the WE was grounded with Si–C paper (300–800 grit), washed with fresh double distilled water, degreased with acetone, and dried. A Gamry REF 3000 potentiostat/galvanostat/Zra instrument was used for the measurements and the Echem Analyst 7.8.2 program for analysis, fitting and graphing of the data.

### 2.4 Surface examination by SEM and XPS

Scanning electron microscopy (SEM) and X-ray photoelectron spectroscopy (XPS) were used to examine the appearance of the mild steel surface, and therefore the element properties after immersion in the corrosive solution for 24 h with and without the addition of *p*-TSAE and *p*-TSAH molecules. SEM tests were done on an SEM, JOEL, JSM-T20, Japan instrument with an acceleration voltage of 30.00 kV and a magnification of 1000 times. XPS was carried out on a Scienta Omicron Nanotechnology instrument, Germany.

### 2.5 Theoretical models

The geometries of the synthesized molecules in three dimensions were built using the Gaussview 6 (ref. 27) cartesian coordinates builder and were optimized following the convergence criteria of the Gaussian 09 software package.^28^ The theoretical procedure included Becke-style 3-parameter density functional theory using the Lee–Yang–Parr correlation functional, B3LYP, which was of reasonable computational cost for the specified molecules.^29–31^ The convergence was reached at a maximum force of 0.1 × 10^−5^ and displacement of 1.6 × 10^−5^, while the thresholds for energy and displacement were 45 × 10^−5^ and 180 × 10^−5^, respectively. The molecular orbitals were represented by the Gaussview 6 MO editor to accurately approximate the exact orbital densities. The frontier molecular orbital surfaces were adjusted to an isovalue of 0.02 and density of 0.0004 and presented as mesh surfaces using the molecular editor of Gaussview 6.^27^ The natural bond orbital surfaces were obtained using NBO version 3.1 (ref. 32) incorporated in the Gaussian 09 package. The NBO surfaces were extracted using the same edits as for the frontier molecular orbitals to accurately compare them. The parameters accounting for global reactivity were calculated using the HOMO and LUMO eigenvalues obtained from the DFT calculation.^33^ The structures were then used in a single point calculation to obtain the molecule energies, charges and populations using Hirshfeld surface analysis, in which there was a neutral molecule, singly-charged cation and singly-charged anion, in order to perform local reactivity analysis using Fukui functions. The closed shell model in Gaussian 09 forces each electron pair, in neutral molecules, into a single spatial orbital, while switching to the open shell model, to treat electrons spin up and spin down, in charged molecules.

Monte Carlo (MC) simulation was used to investigate the interactions of *p*-TSAE and *p*-TSAH with the mild steel surface. The adsorption locator module of the Materials Studio 2017 software was utilized to examine how these molecules interacted with the Fe (110) surface.^34^ The COMPASS force field was used in all system adsorption computations. To verify that a sufficient depth was obtained, a crystal of Fe (110), with an edge of 30 Å, was built. This crystal was subsequently magnified to form a supercell (11 × 11). The optimal structures of *p*-TSAE and *p*-TSAH were investigated in order to find the most stable adsorption configurations on the Fe (110) surface in the neutral and protonated forms in the gas phase and in the presence of water and HCl molecules.

## 3. Results and discussion

### 3.1 Synthesis and characterization of inhibitors

#### 3.1.1 Synthetic route of the ester (*p*-TSAE, 8) and hydrazide (*p*-TSAH, 9)

As shown in Scheme 1, our target sulfonamide ethyl ester (*p*-TSAE, 8) has been previously synthesized *via* modern organic transformations that were mediated by Pd catalysis according to Shekhar *et al.*'s paper^24^ and Cao *et al.*'s work.^25^ The corresponding methyl ester was also recently synthesized *via* a two-step conventional method that was documented in 2020 by Hamdani group's,^26^ who further converted it to our target hydrazide (*p*-TSAH, 9) with 78% yield.^26^ In our large scalable approach that depends on the Hinsberg reaction, commercially available starting material 4-toluenesulfonyl chloride (tosyl chloride, 5) was directly coupled with ethyl 4-aminobenzoate (6) in dichloromethane (DCM) containing triethylamine (Et_3_N) to afford the desired sulfonamide ethyl ester (*p*-TSAE, 8) in excellent yield (95%). Then, the pyridine-catalyzed hydrazinolysis of 8 with 100% hydrazine hydrate was carried out in refluxing ethanol to generate the corresponding acyl hydrazide derivative (*p*-TSAE, 8) in high yield (91%). The target ester 8 and its acyl hydrazide 9 were characterized by physical properties, FT-IR, NMR, and mass spectrometry analysis.

#### 3.1.2 FT-IR spectral analysis

**Fig. 1:**
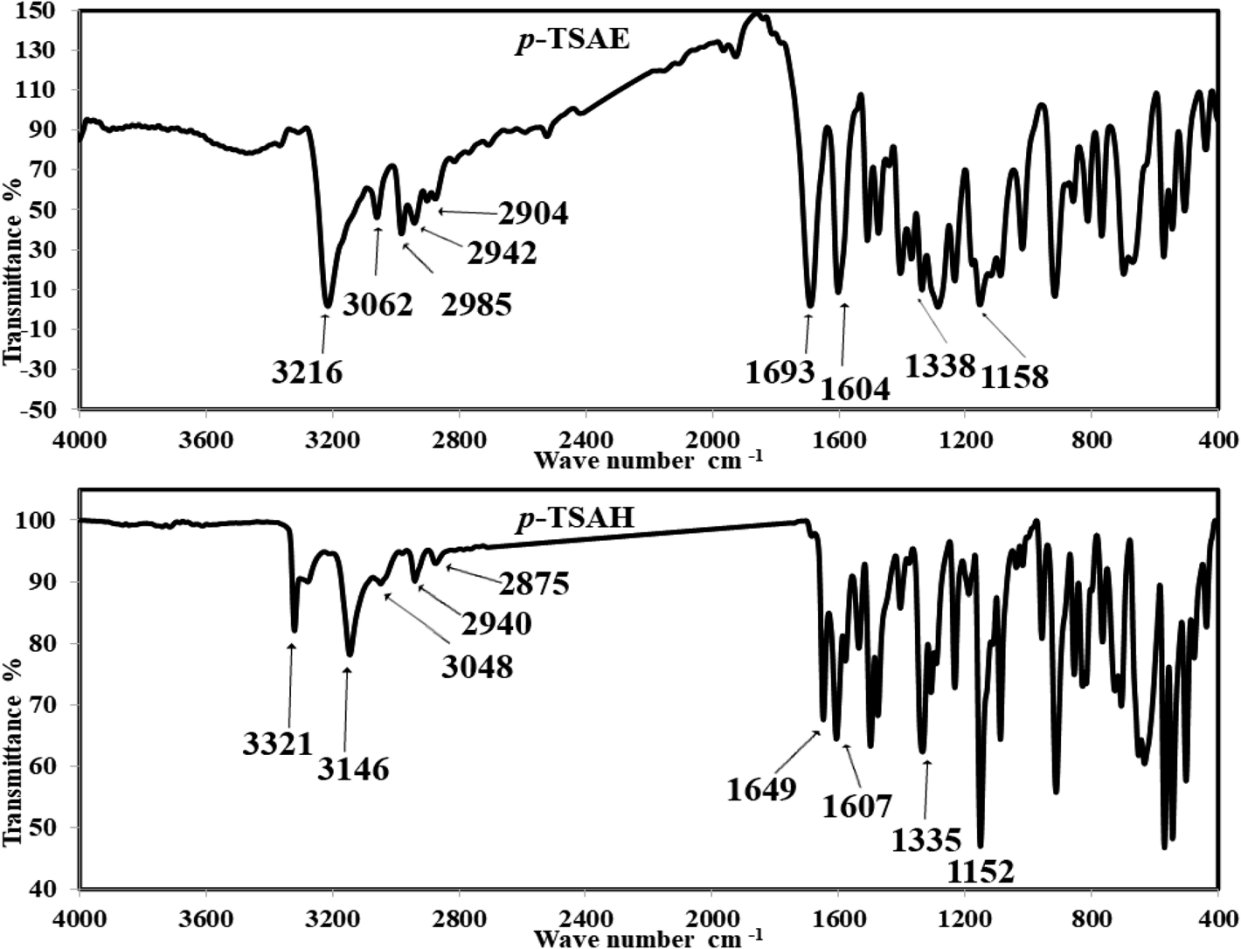
FT-IR spectra of the ester 8 (*p*-TSAE) and the acyl hydrazide 9 (*p*-TSAH).

The mid-infrared spectrum of 8, recorded in KBr, shows a strong band at 3216 cm^−1^ corresponding to the N–H stretching vibration,^26^ which proves the sulfonamide formation. The sp^2^-(aromatic stretching) and sp^3^-CH (aliphatic stretching) valence vibrations are assigned to 3062–2904 cm^−1^. Additionally, strong bands in the 1693 and 1604 cm^−1^ regions can be attributed to C=O and C=C stretching vibrations. The O=S=O stretching bands are observed at 1338 and 1158 cm^−1^ (ref. 26) (Fig. 1). The solid mode IR spectrum of acyl hydrazide 9 shows new frequencies, one of which is a slightly split medium band at 3321 and 3279 cm^−1^ due to NH_2_ vibration, and the other a medium band at 3146 cm^−1^, arising from N–H stretching. Moving to the double bond region, we detect two medium bands at 1649 and 1607 cm^−1^, which are probably attributable to the acyl hydrazide C=O and aromatic C=C moieties. Upon comparison with ester 8, O=S=O shows two similar bands with slight differences at 1335 and 1152 cm^−1^ (ref. 26) (Fig. 1).

#### 3.1.3 NMR spectroscopic analysis

**Fig. 2:**
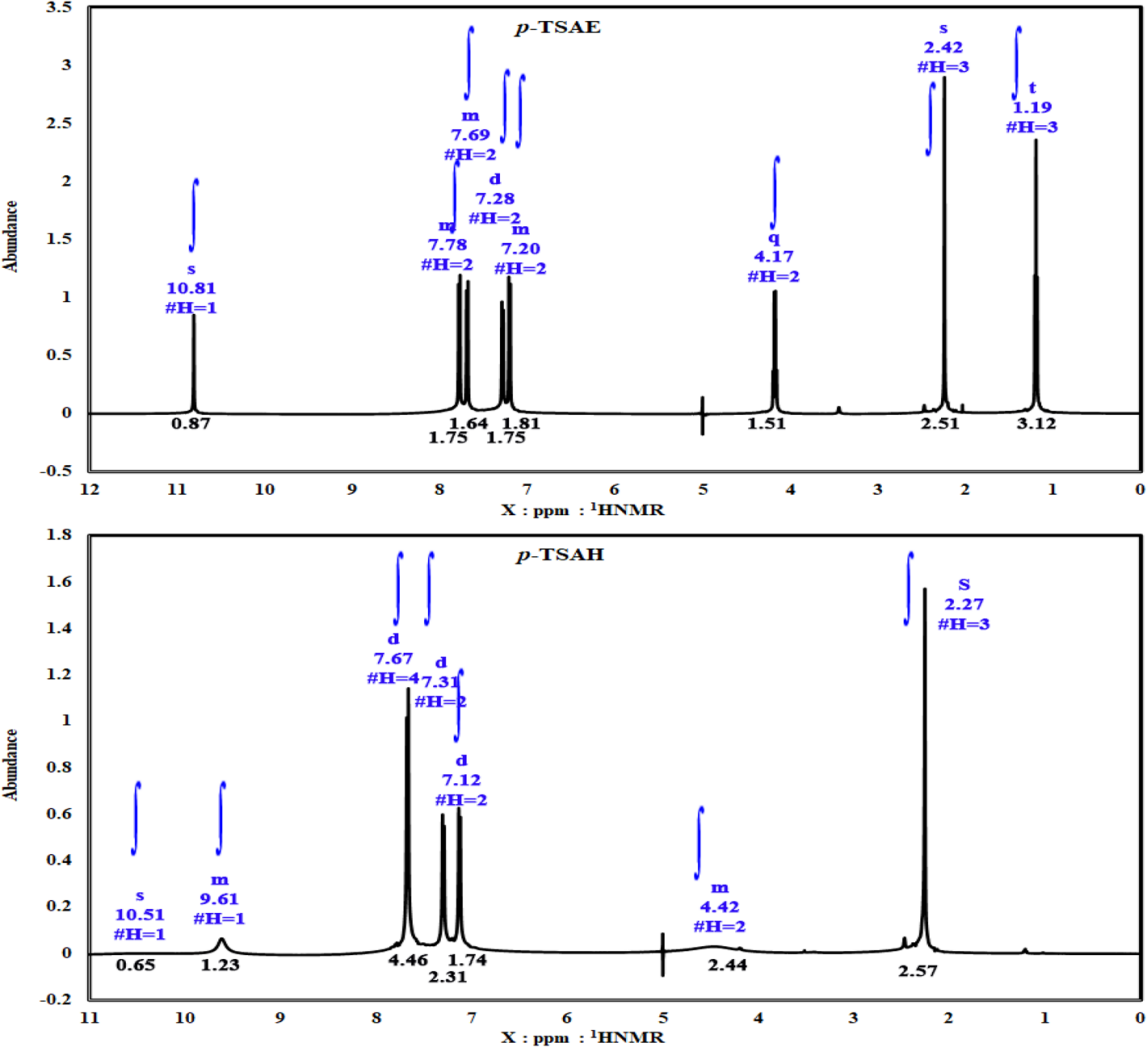
^1^H NMR spectra (500 MHz, DMSO-*d*_6_) of the ester 8 (*p*-TSAE) and the acyl hydrazide 9 (*p*-TSAH).

**Fig. 3:**
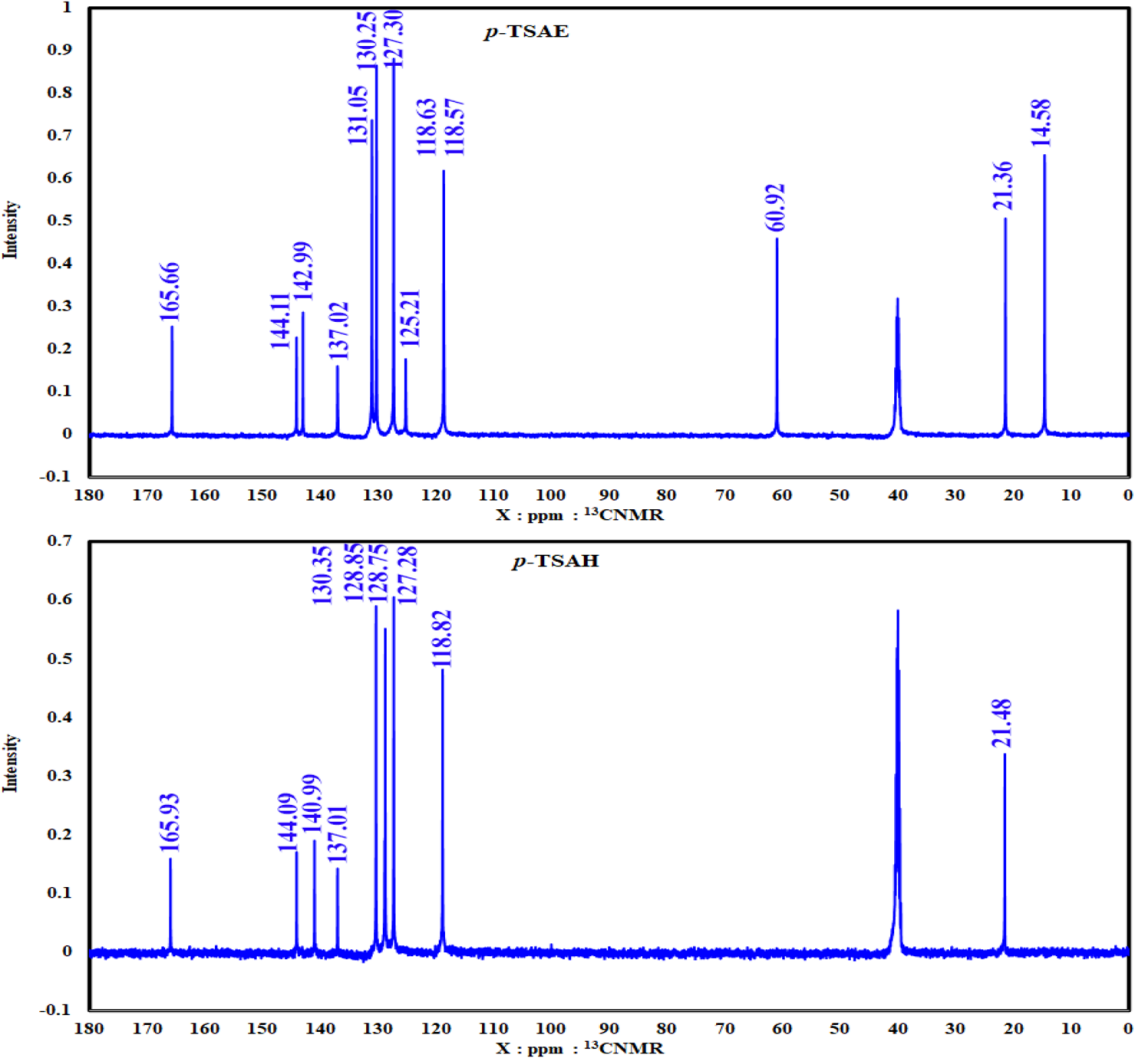
^13^CNMR spectra (125 MHz, DMSO) of the ester 8 (*p*-TSAE) and the acyl hydrazide 9 (*p*-TSAH).

The ^1^H NMR spectrum of sulfonamide ethyl ester 8 (Fig. 2), recorded in DMSO-*d*_6_ inspected from left to right first reveals a singlet ^1^H resonance at *δ* 10.81 ppm, which is attributable to the N***H*** proton,^24^ proving the formation of a sulfonamide linkage. In the aromatic region, eight ^1^H chemical shifts are generated from *δ* 7.78 to 7.20 ppm, which correspond to the typical spin AA′XX′ pattern of two 1,4-disubstituted phenyl rings. Moving to the upfield region, we find three proton resonances. Two among them are quartets (q) one at *δ* 4.17 ppm, and a triplet (t) signal at *δ* 1.19 ppm, which clearly belongs to the ester group (*J* = 7.1 Hz). The remaining singlet signal at *δ* 2.24 ppm with the highest intensity must belong to aromatic methyl protons.^24^ In the clear carbon NMR analysis of 8 (Fig. 3), a ^13^C resonance at *δ* 165.66 ppm can be directly assigned to the carbonyl ester group.^24,25^ The non-protonated and CH aromatic carbon signals ranging from *δ* 144.11 to 118.57 ppm are also shown. In the aliphatic region, methylene ester and its methyl carbon show resonances at *δ* 60.92 and 14.58 ppm, respectively, whereas the aromatic methyl carbon is seen as expected at *δ* 21.36 ppm.^24,25^

As shown in the 1D ^1^H NMR spectrum of acyl hydrazide derivative 9 (Fig. 2), the sulfonamide N***H*** is slightly shielded at *δ* 10.51 ppm and a new peak corresponding to the hydrazide group –CON***H***NH_2_ is present at *δ* 9.61 ppm, confirming the successful hydrazinolysis step.^26^ The typical AA′XX′ pattern of two 1,4-disubstituted benzene rings is observed between *δ* 7.67 and 7.12 ppm. Prior to the aliphatic region, the NH_2_ group is observed as a broad singlet at *δ* 4.42 ppm.^26^ The absence of a peak corresponding to an ethyl ester group is indicative of the complete conversion of 8 to 9*via* the hydrazinolysis reaction. As expected, the aromatic methyl group is shown as a singlet signal with the highest intensity at *δ* 2.27 ppm. Additionally, the well-analyzed ^13^C NMR spectrum of 9 (Fig. 3), reported for the first time, confirms the absence of the ethyl ester group, and shows the carbonyl chemical shift at *δ* 165.93 ppm, whilst the methyl of the tosyl fragment is shown at *δ* 21.48 ppm.

#### 3.1.4 EI mass spectrometry

**Fig. 4:**
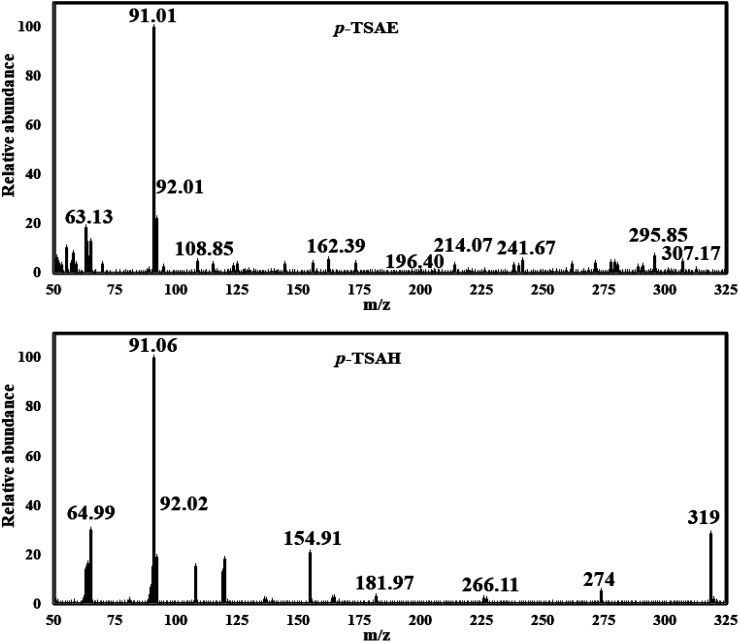
EI-MS spectrum of the ester 8 (*p*-TSAE) and the acyl hydrazide 9 (*p*-TSAH).

**Scheme 2:**
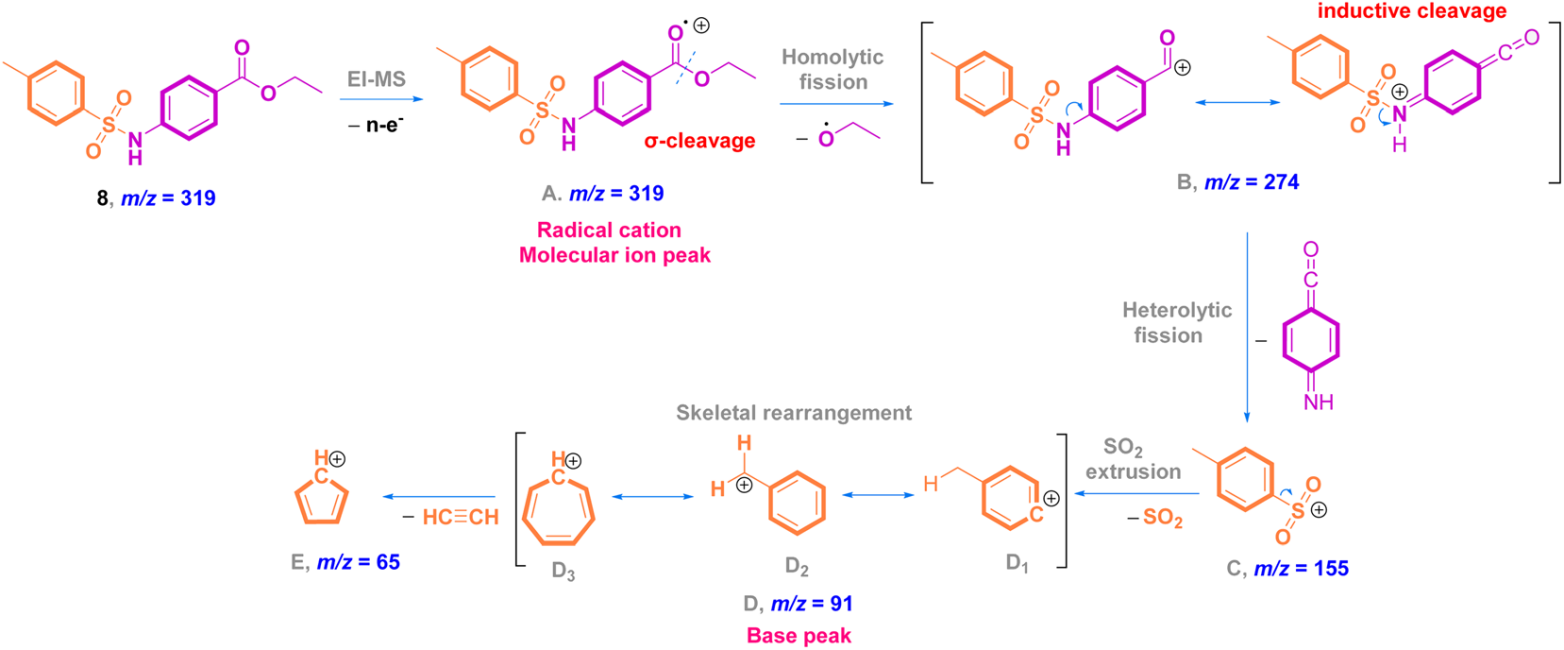
Proposed fragmentation pathway of methyl-sulfonamide ester 8.

In the EI mass spectrum of 8, the tallest basic peak at *m*/*z* = 91 (100%)^25^ is assigned to the formation of the *p*-tolyl fragment ion. Another peak with a relative intensity of 20% is generated at *m*/*z* = 155 due to the formation of the tosyl ion. Additionally, in the mass stick diagram of 8, the line generated by the heaviest ion flowing *via* the machine at *m*/*z* = 319 [M]^+^ (30%) is related to the parent ion (Fig. 4). Concerning the fragmentation pattern of methyl-sulfonamide ester 8 (Scheme 2), radical cation A at *m*/*z* = 319 undergoes homolysis *via* sigma bond cleavage (σ-cleavage) of the carbon–oxygen (C–O) bond giving rise to the fragment ion B at *m*/*z* = 274 after the removal of an ethoxy radical (C_2_H_5_O˙) of 45 Da. The radical migrates from the alpha carbon to the carbonyl oxygen radical that has a strong tendency for electron pairing, which acts as a driving force of this fragmentation type (radical site-initiated fragmentation). Upon the further fragmentation of B by heterolytic dissociation of the S–N bond,^35^ a common fragment ion C of aromatic sulfonamides at *m*/*z* = 155 is formed.^36,37^ The inductive effect of the positive nitrogen results in the fragmentation step (charge site-initiated cleavage). In addition, the extrusion of SO_2_ (loss of 64 Da)^37^ through the direct heterolytic cleavage of the C–S bond affords the most common *p*-tolyl fragment ion D_1_ at *m*/*z* = 91 with the highest abundance (100%, base peak). Further skeletal rearrangement leads to the conversion of D_1_ to D_2_ and D_3_ ions, which affords the last fragment ion E at *m*/*z* = 65 due to the expulsion of acetylene gas of 26 Da.

**Scheme 3:**
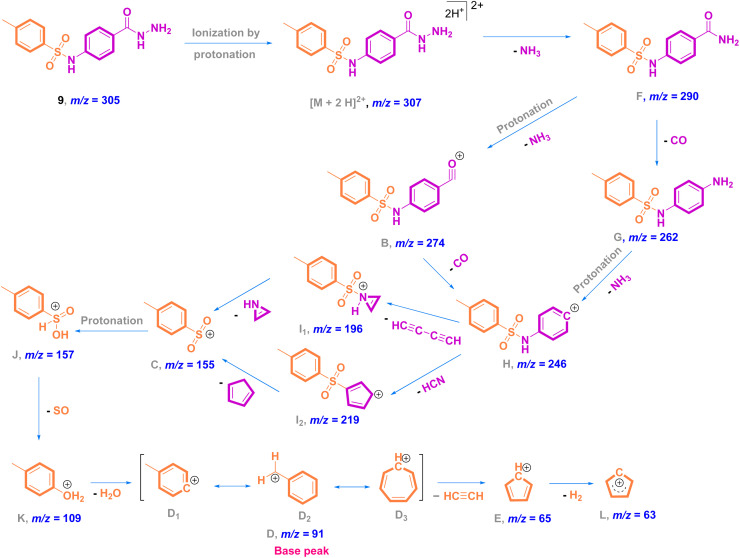
Proposed fragmentation pathways of methyl-sulfonamide hydrazide 9.

Mass analysis supported the chemical structure of 9 (non-reported) with a molecular ion peak at *m*/*z* = 307 [M + 2]^+^ (5%) and the *p*-tolyl fragment ion as the same base peak as that of 8 at *m*/*z* = 91 (100%) (Fig. 4). Ionization occurs by protonation of 9 to form the molecular ion peak at *m*/*z* = 307. Protonated hydrazide [M + 2H]^2+^ is fragmented to afford the corresponding amide F at *m*/*z* = 290 by elimination of NH_3_ (loss of 17 Da).^38^ Additional fragmentation of F*via* inductive cleavage of the C–N bond yields the corresponding acylium ion B at *m*/*z* = 274 after the loss of NH_3_ of 17 Da.^39^ Additionally, F can undergo decarbonylation (loss of CO, 28 Da) to produce amine G at *m*/*z* = 262. Both B and G may further dissociate to exhibit the prospective phenyl cation H at *m*/*z* = 246. Fragmentation of H after the neutral loss of a diacetylene gas molecule of 50 Da resulted in the formation of azirine ion I_1_ at *m*/*z* = 196. Additionally, H can be fragmented by the loss of HCN of 27 Da to produce another possible ion I_2_ at *m*/*z* = 219. Both fragment cations I_1_ and I_2_ can further disassociate to form the known ion C at *m*/*z* = 155,^36,37^ which upon protonation converts to cation J. The rearrangement of J drives the elimination of SO and the formation of an ion at *m*/*z* = 109,^36^ which dehydrates to generate the base peak at *m*/*z* = 91 (Scheme 3).^37^

### 3.2 Electrochemical measurements

#### 3.2.1 Open circuit potential (OCP)

**Fig. 5:**
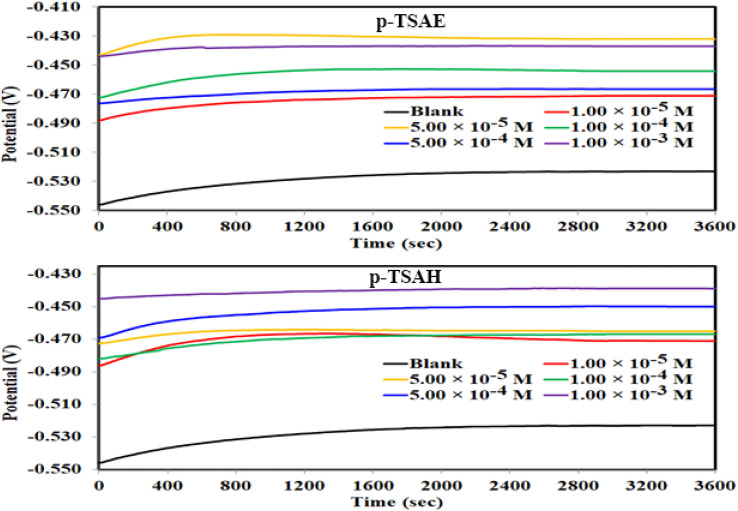
OCP–time curves for mild steel in 1.0 M HCl solution with and without different concentrations of *p*-TSAH and *p*-TSAE compounds at 30 °C.

The corrosion potential, simply the potential difference between the working and reference electrode, was probed *versus* time to obtain a steady state for the investigated systems prior to the following electrochemical experiments. At a steady state, the rate of the corrosion reactions is assumed to be constant. A one-hour experiment was conducted at a temperature of 303.15 K for this purpose. The resultant potential *vs.* time curves for the mild steel/1.0 M HCl/(*p*-TSAH or *p*-TSAE) systems are presented in Fig. 5. It can be inferred from the curves that the potential through all the solutions of *p*-TSAH or *p*-TSAE reached minimal fluctuations (±5 mV or less) almost at the same time. While measuring the corrosion potential is an easy task for a potentiostat, it guarantees the accuracy of the following EFM, PDP and EIS measurements. Moreover, upon adding concentrations of *p*-TSAH or *p*-TSAE inhibitors to the blank solutions, a positive shift in the potential can be noticed, implying the adsorption of these molecules to the mild steel surface and blocking of the surface corroded active sites.^40^

#### 3.2.2 Electrochemical frequency modulations

**Fig. 6:**
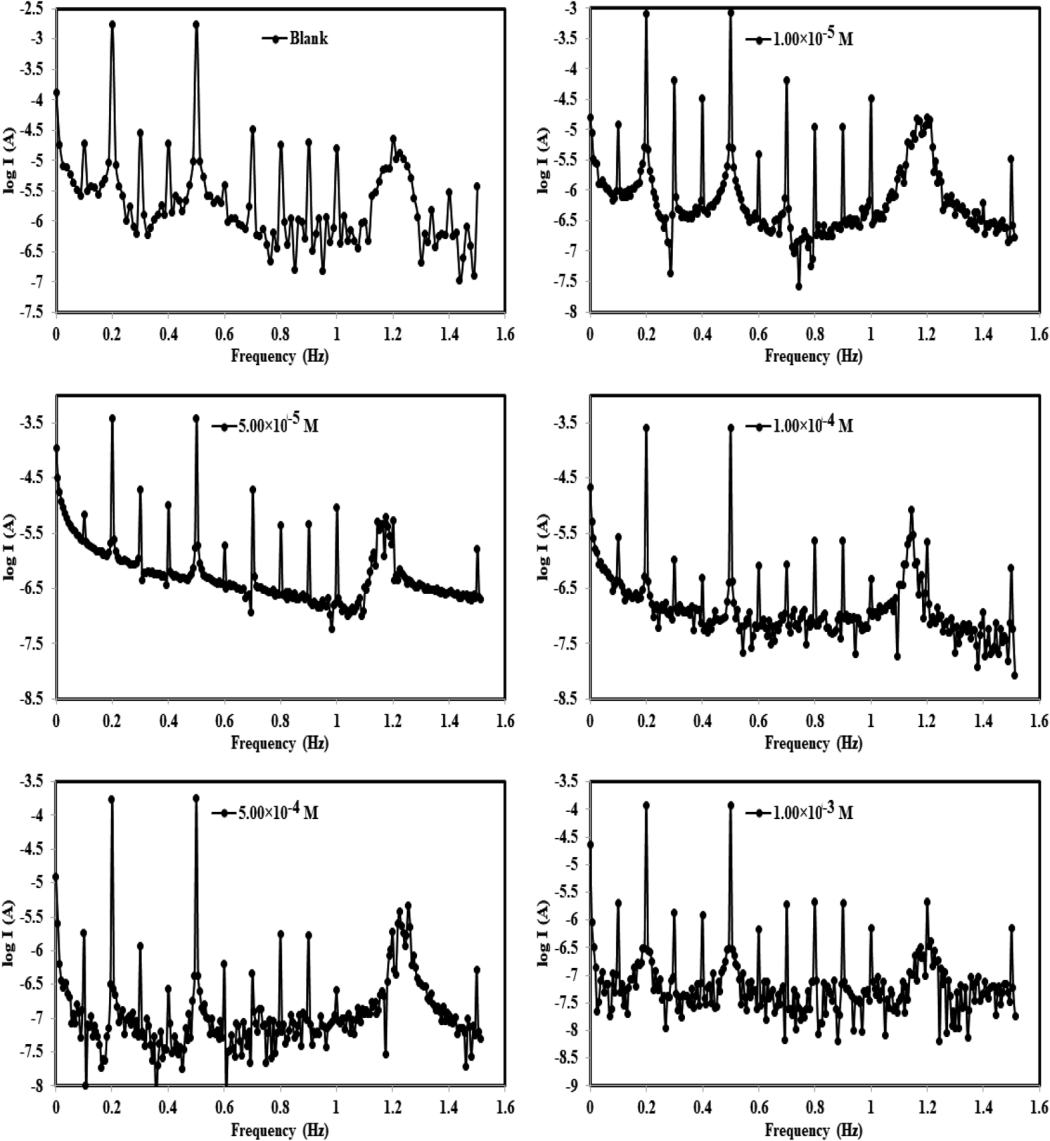
Intermodulation spectra for steel in 1.0 M HCl in the absence and presence of different concentrations of the *p*-TSAH compound at 30 °C.

After the OCP curves of the tested solutions had achieved stability, EFM was immediately carried out to evaluate the corrosion inhibition behavior of the *p*-TSAH and *p*-TSAE molecules for the mild steel surface in a 1.0 M HCl solution. The EFM technique has several advanced characteristics, including being a non-destructive and reliable corrosion testing technique.^41^Fig. 6 shows the intermodulation spectra for mild steel immersed in the corrosive medium in the absence and presence of *p*-TSAH molecules at a temperature of 303.15 K. These spectra show two intense and characteristic peaks at 0.2 and 0.5 Hz related to the selected excitation frequencies, besides other low intensity peaks related to the harmonic sum, differences, and multiples of the selected two excitation frequencies.^42^ The current responses at the two selected excitation frequencies determine the corrosion rate, and therefore the inhibition efficiencies. The extracted electrochemical parameters from the EFM measurements are shown in Table 2. The reliability of the obtained data was checked through the values of the causality factors. It is shown in Table 2 that the empirical values of CF-2 and CF-3 are close to their theoretical values (2 and 3).^43^ The EFM spectra for the inhibited solutions are observed in more negative current areas than the corrosive 1 M HCl solution, so the presence of *p*-TSAH and *p*-TSAE molecules decreases the current flow between the steel surface and the reference electrode. The highest value of *i*_corr_ being in the inhibitor free solution with respect to the solutions containing *p*-TSAH or *p*-TSAE molecules indicated that corrosive ions such as Cl^−^ are more activated and a high corrosion rate occurred. However, after the addition of *p*-TSAH or *p*-TSAE molecules, the corrosion current densities are reduced, and the steel surface protection is enhanced. This is related to the adsorption of *p*-TSAH or *p*-TSAE*via* the organic hetero atoms (S, N and O) and the aromatic moieties, blocking the corrosion active sites and slowing down the current flow.^44^ The values of the corrosion current are 2791 μA cm^−2^ for the blank 1 M HCl solution, 1453 μA cm^−2^ for the *p*-TSAE compound and 1287 μA cm^−2^ for the *p*-TSAH compound at the lowest concentration of 1.00 × 10^−5^ M, so the corrosion of steel is decreased by the addition of *p*-TSAH or *p*-TSAE molecules. The successive addition of the inhibitor molecules gradually decreases the current density. As an example (for the *p*-TSAH molecule), *i*_corr_ is 599.5 μA cm^−2^ at 5.00 × 10^−5^ M, 463.6 μA cm^−2^ at 1.00 × 10^−4^ M, 303.5 μA cm^−2^ at 5.00 × 10^−4^ M and 158.6 μA cm^−2^ at 1.00 × 10^−3^ M. This situation is observed also for the corrosion rate, as the highest value was observed for the 1.0 M HCl solution (1275.00 mpy), and this was greatly reduced to 664.10 mpy and 587.90 mpy by the addition of the lowest dose (1.00 × 10^−5^ M) of the studied molecules. The values of *β*_a_ and *β*_c_ are impacted by the addition of *p*-TSAH or *p*-TSAE molecules as evidence of the mixed inhibition performance of these two molecules by the adsorption on both cathodic and anodic active sites. The protective ability of *p*-TSAH and *p*-TSAE were estimated from *i*_corr_ as per the following:^45^1
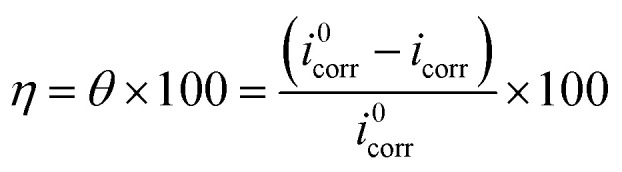
*i*_corr_^0^ = corrosion current density of the blank solution, *i*_corr_ = corrosion current density of any desired concentration of *p*-TSAH or *p*-TSAE molecules.

**Table 2:** Electrochemical kinetic parameters^a^ obtained by the EFM technique for steel in the absence and presence of various concentrations of *p*-TSAE and *p*-TSAH inhibitors in 1.0 M HCl at 30 °C

Inhibitor name	Conc. (M)	*i*_corr_ (μA cm^−2^)	*β*_a_ (mV dec^−1^)	*β*_c_ (mV dec^−1^)	CF-2	CF-3	*k* (mpy)	*θ*	*η*_EFM_%
Blank	—	2791	100.4	113.1	1.763	3.155	1275.00	—	—
*p*-TSAE	1.00 × 10^−5^	1453	99.04	186	2.013	2.853	664.10	0.4794	47.94
	5.00 × 10^−5^	745.4	102.5	131.1	1.989	2.992	340.60	0.7329	73.29
	1.00 × 10^−4^	534.8	116.3	121.3	2.204	3.076	244.40	0.8084	80.84
	5.00 × 10^−4^	380.6	116.5	124	1.992	2.887	173.90	0.8636	86.36
	1.00 × 10^−3^	209	94.66	114.1	1.862	3.131	95.50	0.9251	92.51
*p*-TSAH	1.00 × 10^−5^	1287	81.73	133.6	1.967	3.424	587.90	0.5389	53.89
	5.00 × 10^−5^	599.5	87.95	120.8	1.986	3.061	273.90	0.7852	78.52
	1.00 × 10^−4^	463.6	117.6	120.9	1.982	3.037	211.80	0.8339	83.39
	5.00 × 10^−4^	303.5	111.4	115.1	2.074	3.099	138.70	0.8913	89.13
	1.00 × 10^−3^	158.6	84.18	90.8	1.729	2.975	72.46	0.9432	94.32

a *E*_corr_ is the corrosion potential; *i*_corr_ is the corrosion current density; *β*_a_ and *β*_c_ are Tafel constants for the anode and cathode; *k* is the corrosion rate; *θ* is the surface coverage; *η*_EFM_ is the inhibition efficiency.

The results confirmed that the high protecting ability of the *p*-TSAH and *p*-TSAE molecules approached 94.32% (*p*-TSAH) and 92.51% (*p*-TSAE) at 1.00 × 10^−3^ M. The slight difference in inhibition efficiency between the *p*-TSAH molecule and the *p*-TSAE molecule might be due to the presence of extra nitrogen atoms in the molecular skeleton of the former. This facilitates its adsorption to enhance the protection.

#### 3.2.3 Potentiodynamic polarization measurements

**Fig. 7:**
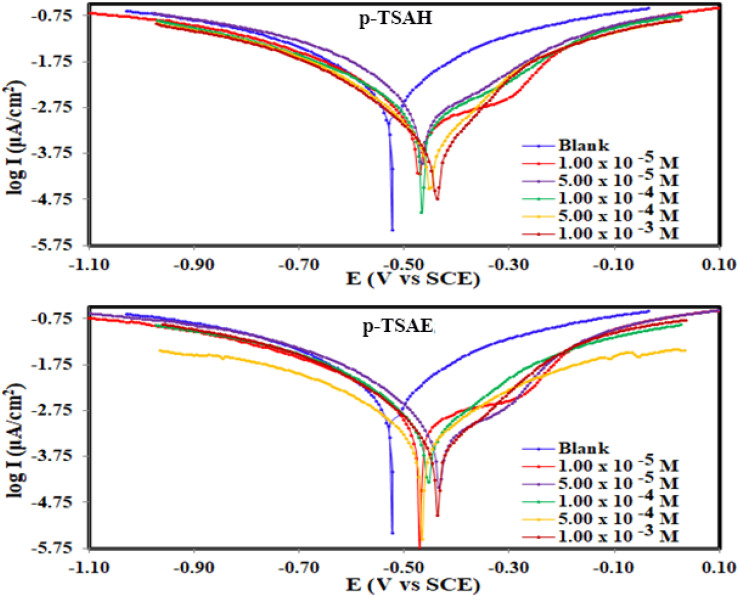
Potentiodynamic polarization curves for the corrosion of steel in 1.0 M HCl in the absence and presence of different concentrations of *p*-TSAH and *p*-TSAE compounds at 30 °C.

Tafel plots, from the potentiodynamic polarization method, for the corrosion of mild steel in 1.0 HCl solution (blank, with *p*-TSAH and with *p*-TSAE), are presented in Fig. 7. It can be observed through the plots that the polarized curves were affected when the inhibitors were added to the blank solution. A noticeable decrease in the rate of both anodic and cathodic reactions could be inferred from the comparison of the blank *vs.* inhibitor (*p*-TSAH and *p*-TSAE) curves. However, the anodic reactions were altered more noticeably by the presence of *p*-TSAH and *p*-TSAE molecules than the cathodic ones. The latter are in the close vicinity of the curves of the 1.0 M HCl blank solution. Overall, a reduction in the corrosion reactions in both the anodic and cathodic directions could be observed, nevertheless the anodic reactions (signifying mild steel dissolution) were more retarded.^46^ The parallel polarization curves support the idea that the addition of *p*-TSAH or *p*-TSAE does not alter the pathway of the corrosion reaction.

The results derived from the Tafel plots, by fitting and extrapolation of the log current *versus* potential curves back to their intersections, in addition to the inhibition efficiency and surface coverage, are shown in Table 3. The data reveal that the Tafel constants (*β*_a_ and *β*_c_) move to lower values when *p*-TSAH and *p*-TSAE samples are used in the corrosion systems. This suggests that *p*-TSAH and *p*-TSAE molecules contribute to the electrochemical reactions (both cathodic and anodic) through an adsorption mechanism promoting an intact metallic surface.^47^ Accordingly, the addition of *p*-TSAH or *p*-TSAE molecules significantly decreases the corrosion current *i*_corr_; the minimum obtained value is 580 μA cm^−2^ in the case of *p*-TSAH and 710 μA cm^−2^ in the case of *p*-TSAE at 1.00 × 10^−3^ M, Table 3. Correspondingly, *E*_corr_ shifted to more positive values in the presence of *p*-TSAH or *p*-TSAE molecules while the difference, *i.e.* (Δ*E*_corr_ = |*E*_corr_(blank) − *E*_corr_(inhibitor)|), is less than 85 mV in most solutions. Therefore, a mixed type inhibition behavior of *p*-TSAH and *p*-TSAE molecules could be suggested.^48^ Furthermore, the tendency for passivation to take place by *p*-TSAH and *p*-TSAE is a function of *i*_corr_ according to eqn (1), as described in section 3.2.2.

**Table 3:** Electrochemical parameters^a^ for steel dissolution in 1.0 M HCl solution containing different concentrations of the *p*-TSAE and *p*-TSAH inhibitors obtained from polarization measurements at 30 °C

Inhibitor name	Conc. (M)	*E*_corr_ *vs.* SCE (mV)	*i*_corr_ (μA cm^−2^)	*β*_a_ (mV dec^−1^)	*β*_c_ (mV dec^−1^)	*k* (mpy)	Δ*E*_corr_ (mV)	*θ*	*η*_PDP_%
Blank	—	−523	8624	305	333	4137	—	—	—
*p*-TSAE	1.00 × 10^−5^	−471	4050	607.8	572.6	1852	52	0.530	53.02
	5.00 × 10^−5^	−432	2730	526	429.2	1248	91	0.683	68.33
	1.00 × 10^−4^	−454	1670	234.9	249.6	762.6	69	0.806	80.63
	5.00 × 10^−4^	−466	1170	295.1	302.6	536	57	0.864	86.43
	1.00 × 10^−3^	−437	710	183.8	209.4	324.5	86	0.918	91.76
*p*-TSAH	1.00 × 10^−5^	−471	3740	579.1	599.6	1709	52	0.566	56.61
	5.00 × 10^−5^	−465	2550	243.2	243.5	1164	58	0.704	70.42
	1.00 × 10^−4^	−467	1410	219.4	232.1	646	56	0.836	83.64
	5.00 × 10^−4^	−450	843	195.3	221.9	385.1	73	0.902	90.22
	1.00 × 10^−3^	−439	580	177.1	212.8	265	84	0.933	93.27

a *E*_corr_ is the corrosion potential; *i*_corr_ is the corrosion current density; *β*_a_ and *β*_c_ are Tafel constants for the anode and cathode; *k* is the corrosion rate; *θ* is the surface coverage; *η*_p_ is the inhibition efficiency.

According to the data presented in Table 3, the protection abilities, or *η*_PDP_, of *p*-TSAH and *p*-TSAE from the PDP measurements reached maxima of 93.27% (*p*-TSAH) and 91.76% (*p*-TSAE) at 1.00 × 10^−3^ M. The protection ability gradually improved upon the successive addition of either *p*-TSAH or *p*-TSAE molecules as extra molecules were adsorbed, and more of the mild steel surface was covered. The adsorbed organic layers reduced the interaction between the metallic surface and corrosive mineral acid.^49^ Therefore, the presence of several hetero atoms (S, N and O) in addition to donor–acceptor interactions from the aromatic conjugation π-systems to the empty d-shell of the mild steel facilitated the adsorption and increased the protection ability.

#### 3.2.4 Electrochemical impedance spectroscopy

**Fig. 8:**
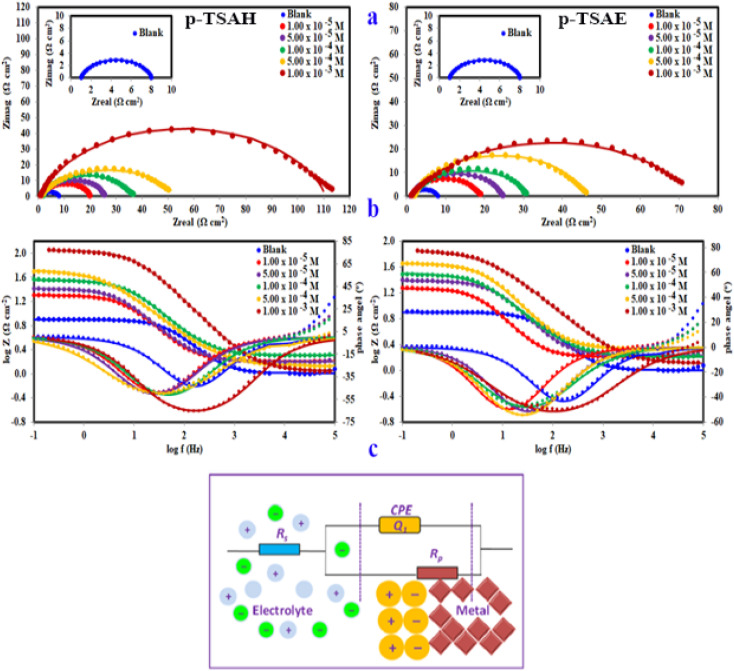
(a) Nyquist and (b) Bode modulus, phase angle plots (the dots show experimental results and the lines show fitted data), and (c) the equivalent circuit model used to fit the EIS data for steel in 1.0 M HCl in the absence and presence of different concentrations of *p*-TSAH and *p*-TSAE compounds at 30 °C.

To highlight the mechanism of the electrochemical process that takes place at the mild steel surface, EIS measurements were performed. EIS outcomes for the mild steel/1.0 M HCl/(*p*-TSAH or *p*-TSAE) systems are presented in Fig. 8, which includes Nyquist and Bode plots and the corresponding equivalent circuit model. Furthermore, corresponding EIS fitting data are collected in Table 4. Both the Nyquist and Bode plots are characterized by a single time constant that is related to the corrosion as a charge transfer process taking place at the metallic surface.^50^ The semi-circles obtained for the Nyquist plots are imperfect capacitive loops, at the high frequency regions, which could be attributed to metallic surface heterogeneity and roughness caused by exposure to the aggressive mineral acid. In addition, it can be seen in Fig. 8 that the semi-circle is single and depressed in the case of both the blank and the inhibited solutions. Capacitive semicircles corresponding to charge-transfer and adsorption processes are often depressed, therefore, it can be concluded that the charge transfer pathway controls both the corrosion and the inhibition reactions, while the used inhibitors control these reactions through adsorption to the metal surface. Additionally, the diameter of the semi-circle is gradually increased by the successive addition of *p*-TSAH or *p*-TSAE molecules because of their adsorption on the metallic surface and the formation of a layer that protects it from corrosion. Adsorption considerations will be discussed in detail in a separate section.

**Table 4:** EIS parameters^a^ for the corrosion of steel in 1.0 M HCl in the absence and presence of different concentrations of *p*-TSAE and *p*-TSAH inhibitors at 30 °C

Inhibitor	Conc. (M)	*R*_s_ (*R*_u_) (Ω cm^2^)	*R*_ct_ (*R*_p_) (Ω cm^2^)	*Q* (μ Ω ^−1^ s^*n*^ cm^−2^)	*n*	*C*_dl_ (μF cm^−2^)	chi squared	*S*	*α*°	*θ*	*η*_*z*_ (%)
Blank	—	1.082	6.884	478.50	0.8836	225.363	2.25 × 10^−2^	−0.365	−42.07	—	—
*p*-TSAE	1.00 × 10^−5^	1.612	17.15	366.70	0.8980	267.822	6.60 × 10 ^−3^	−0.469	−49.87	0.5986	59.86
	5.00 × 10^−5^	1.688	22.94	154.90	0.8780	97.423	6.83 × 10 ^−3^	−0.557	−51.01	0.6999	69.99
	1.00 × 10^−4^	1.628	30.30	240.30	0.7819	115.712	7.86 × 10 ^−3^	−0.515	−47.67	0.7728	77.28
	5.00 × 10^−4^	2.169	43.48	75.59	0.8570	42.753	3.34 × 10 ^−3^	−0.657	−54.69	0.8417	84.17
	1.00 × 10^−3^	1.264	72.26	123.00	0.7149	18.702	1.48 × 10^−3^	−0.580	−50.23	0.9047	90.47
*p*-TSAH	1.00 × 10^−5^	1.602	18.65	201.30	0.8670	121.667	6.78 × 10 ^−3^	−0.420	−48.79	0.6309	63.09
	5.00 × 10^−5^	1.582	24.6	218.20	0.8389	124.431	6.95 × 10 ^−3^	−0.429	−50.02	0.7202	72.02
	1.00 × 10^−4^	1.996	34.33	109.00	0.8303	55.692	5.04 × 10 ^−3^	−0.517	−50.63	0.7995	79.95
	5.00 × 10^−4^	1.257	54.29	479.00	0.7459	138.167	1.15 × 10^−3^	−0.502	−50.18	0.8732	87.32
	1.00 × 10^−3^	1.116	109.8	377.50	0.8446	210.160	7.65 × 10^−4^	−0.746	−65.77	0.9373	93.73

a *R*_s_ = solution resistance, *R*_ct_ = charge transfer resistance, *Q*, *n* = variation parameters, *C*_dl_ = double layer capacitance, *θ* = surface coverage, *η*_*z*_ = inhibition efficiency.

The equivalent circuit proposed for the studied systems is a physical model which demands that the model's components are attributed to a physical process in the electrochemical corrosion reaction. Accordingly, the proposed circuit model in Fig. 8 was built up using three components: two resistors, one accounting for the solution resistance (*R*_s_) and the other accounting for the charge transfer resistance (*R*_ct_) in addition to a component with a constant phase. In general, the impedance of a constant phase element CPE (*Z*_CPE_) can be determined using the following equation:^51^

2 *Z*_CPE_ = [*Q*(*jω*)^*n*^]^−1^

*Q* = proportionality factor; *ω* = angular frequency; *j* = −1, and *n* = variation parameter (−1 ≤ *n* ≤ +1). If *n* = 0, CPE = pure resistor; *n* = −1, CPE = inductor; *n* = +1, CPE = pure capacitor.

While a double-layer capacitor is expected to proceed ideally, EIS experiments often do not behave ideally. In such a case, the double-layer capacitor in a real cell acts like a constant phase element (CPE).^52^

The capacitance of the double layer could then be determined by the following relationship:

3 *C*_dl_ = [*QR*_ct_^1−*n*^]^1/*n*^

The *C*_dl_ reduction (Table 4) in the presence of *p*-TSAH or *p*-TSAE could be attributed to a decreased local dielectric constant and increased double layer thickness due to the replacement of H_2_O molecules adsorbed on the metallic surface by the organic inhibitors through the active sites.^53^ The protecting ability is related to the charge transfer resistance *R*_ct_ by the relationship:4
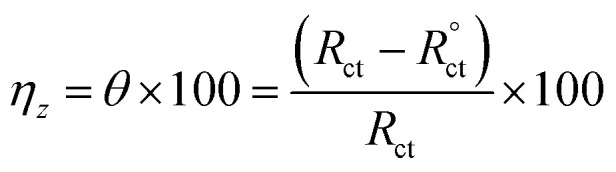


When the capacitance goes down, in contrast, the impedance goes up when the connection is in series. This is a consequence of the inverse relationship between the capacitance and impedance. It can be seen in Table 4 that *R*_ct_ gradually increases upon the addition of *p*-TSAH or *p*-TSAE inhibitors to reach maxima of 109.8 Ω cm^2^ (*p*-TSAH) and 72.26 Ω cm^2^ (for *p*-TSAE) at 1.00 × 10^−3^ M. The corresponding protection abilities at the same concentration are 93.73% for *p*-TSAH and 90.47% for *p*-TSAE. Overall, the EIS electrochemical outcomes support both the PDP and EFM results, which in turn promote the ability of both *p*-TSAH and *p*-TSAE molecules as candidates for effective corrosion inhibition.

Despite the limitless informative outcomes of the Nyquist Plots, they have one major shortcoming in that they cannot give any information about the frequency perturbation within the system under study. Another common representation of EIS data is the Bode plot, in which the frequency is plotted against both the absolute values of the impedance and the phase-shift (Fig. 8b). This representation provides much information about the interfacial organic layer, *p*-TSAH or *p*-TSAE molecules, adsorbed between the metallic surface and the corrosive mineral acid, and contains a single time constant that corresponds to the electrical double layer at the interface. For the blank solution curve in Fig. 8, the phase angle diverges from the standard value for a pure capacitor, which is −90°. This could be a result of frequency scattering due to the surface inhomogeneity caused by acid attack. On the other hand, the addition of either *p*-TSAH or *p*-TSAE molecules shifts the angles to more negative values away from those of the blank solution as an indication of enhanced capacitive behavior and that the surface becomes smoother upon the formation of the adsorbed organic layer. As shown in Fig. 8 and Table 4, the maximum angle deviation is −65.77° for *p*-TSAH and −50.23° for *p*-TSAE at 1.00 × 10^−3^ M. Moreover, the slopes at the middle frequencies shift much closer to the standard value of a pure capacitor of −1 (ref. 54) in the presence of both *p*-TSAH and *p*-TSAE molecules (Table 4).

### 3.3 Adsorption considerations

**Fig. 9:**
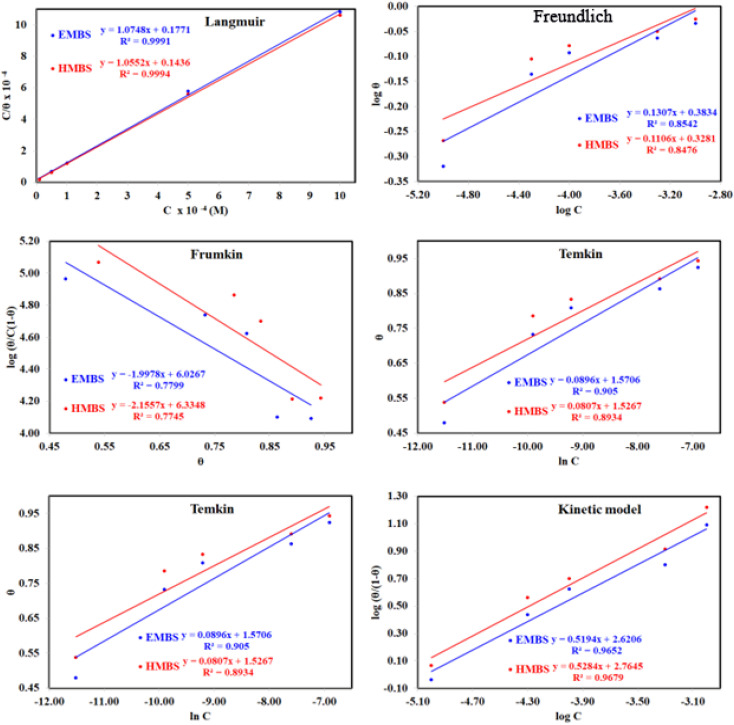
The different adsorption models for *p*-TSAH and *p*-TSAE compounds on the mild steel surface in 1.0 M HCl at 30 °C.

Adsorption isotherms are widely used to investigate the adsorption and protection mechanisms at the mild steel/1.0 M HCl interface. Several traditional adsorption isotherm equations were utilized to fit the results obtained from the EFM measurement of *p*-TSAH and *p*-TSAE molecules.^1^ The fitting data for the six adsorption models are shown in Fig. 9 and Table 5. The quality of the fitting is measured by *R*^2^ values that are close to unity. According to the *R*^2^ values, the quality of the fitting is in the order Langmuir > kinetic–thermodynamic > Flory–Huggins > Temkin > Freundlich > Frumkin. Therefore, the Langmuir model was found to be the most appropriate for the chosen EFM results. This isotherm considers that the metallic adsorbent surface is uniform (the active adsorption sites are equivalent), there is no interaction between the adsorbate (inhibitor) molecules, and the adsorption process occurs by the exchange of single adsorbed water molecules by single inhibitors.^55^ This isotherm is expressed as:^56^5
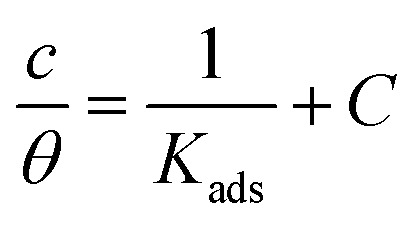


**Table 5:** Adsorption isotherm models of the inhibitors with values of *R*^2^, slopes, intercepts, *K*_ads_, and Δ*G*_ads_ obtained by using data from EFM measurements^a^

Adsorption isotherm model	Linear form equation	Inhibitor	Slope	Intercept	*R* ^2^	*K*_ads_, M^−1^	Δ*G*_ads_, kJ mol^−1^
Freundlich	log *θ* = log *K* + 1/*n* log *C*	*p*-TSAE	0.13066	0.38343	0.85418	2.4178	−12.34
		*p*-TSAH	0.11061	0.32809	0.84755	2.1286	−12.02
Langmuir	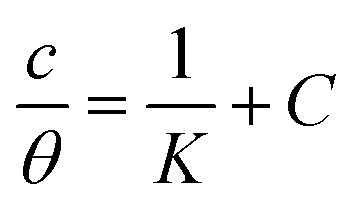	*p*-TSAE	1.07480	0.00002	0.99906	56 463	−37.68
		*p*-TSAH	1.05515	0.00001	0.99937	69 636	−38.21
Frumkin	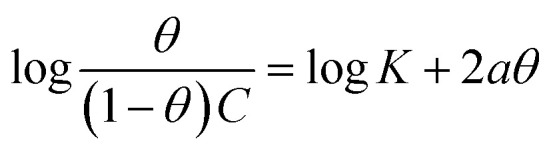	*p*-TSAE	−1.99780	6.02670	0.77986	1.0634 × 10^6^	−45.08
		*p*-TSAH	−2.15565	6.33479	0.77446	2.1617 × 10^6^	−46.86
Temkin	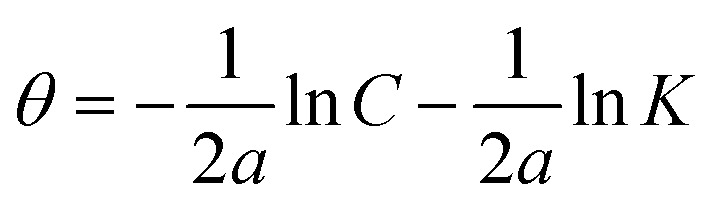	*p*-TSAE	10.10181	−16.72345	0.90502	0.1910	−5.95
		*p*-TSAH	11.07539	−17.87078	0.89339	0.1992	−6.05
Flory–Huggins	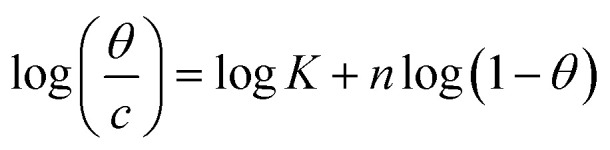	*p*-TSAE	2.16061	5.33216	0.95973	2.1486 × 10^5^	−41.05
		*p*-TSAH	2.06039	5.46038	0.96296	2.8865 × 10^5^	−41.79
Kinetic-thermodynamic	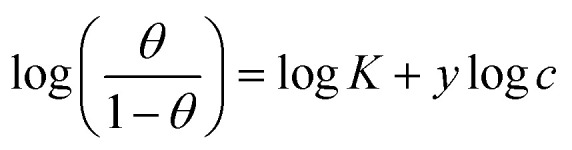	*p*-TSAE	0.51945	2.62065	0.96524	417.4933	−25.32
		*p*-TSAH	0.52835	2.76446	0.96795	581.3738	−26.15

a *R* ^2^ = regression correlation coefficient, *K* = binding constant, *θ* = surface coverage, *c* = concentration.

The adsorptive binding constant (*K*_ads_) is the inverse of the intercept of a *c*/*θ vs. C* plot. The *K*_ads_ values are found to be 69 636 M^−1^ for the *p*-TSAH molecule and 56 463 M^−1^ for the *p*-TSAE molecule. These large values of *K*_ads_ are a good indicator of the strength of the adsorption process, with *p*-TSAH having priority over *p*-TSAE molecules.

The adsorption free energy is estimated from the relationship:^57^

6 Δ*G*_ads_ = −*RT* ln 55.5*K*_ads_

(*R* = 8.314 J mol^−1^ K^−1^, *T* = 303.15 K, [H_2_O] = 55.5 mol L^−1^)

Δ*G*_ads_ values were found to be −38.21 kJ mol^−1^ for the *p*-TSAH molecule and −37.68 kJ mol^−1^ for the *p*-TSAE molecule. The adsorption occurred in a spontaneous way as indicated from the negative values. Δ*G*_ads_ values can be grouped into three categories. The first is for values that are more than −20 kJ mol^−1^, considered as physical adsorption, which describes the electrostatic attraction between the metallic surface and the organic materials. The second category is for values less than −40 kJ mol^−1^, considered as chemical adsorption, which describes electron sharing from the active sites of the organic materials (lone pairs of hetero atoms, π electrons and aromatic moieties) to the empty orbitals of the metal. The third category is for values between −20 kJ mol^−1^ and −40 kJ mol^−1^, considered as mixed type.^58^ Our case matches with the third category as mixed type, but it is greatly shifted towards −40 kJ mol^−1^, so the priority is to chemical adsorption.

### 3.4 Surface analysis

#### 3.4.1 SEM analysis

**Fig. 10:**
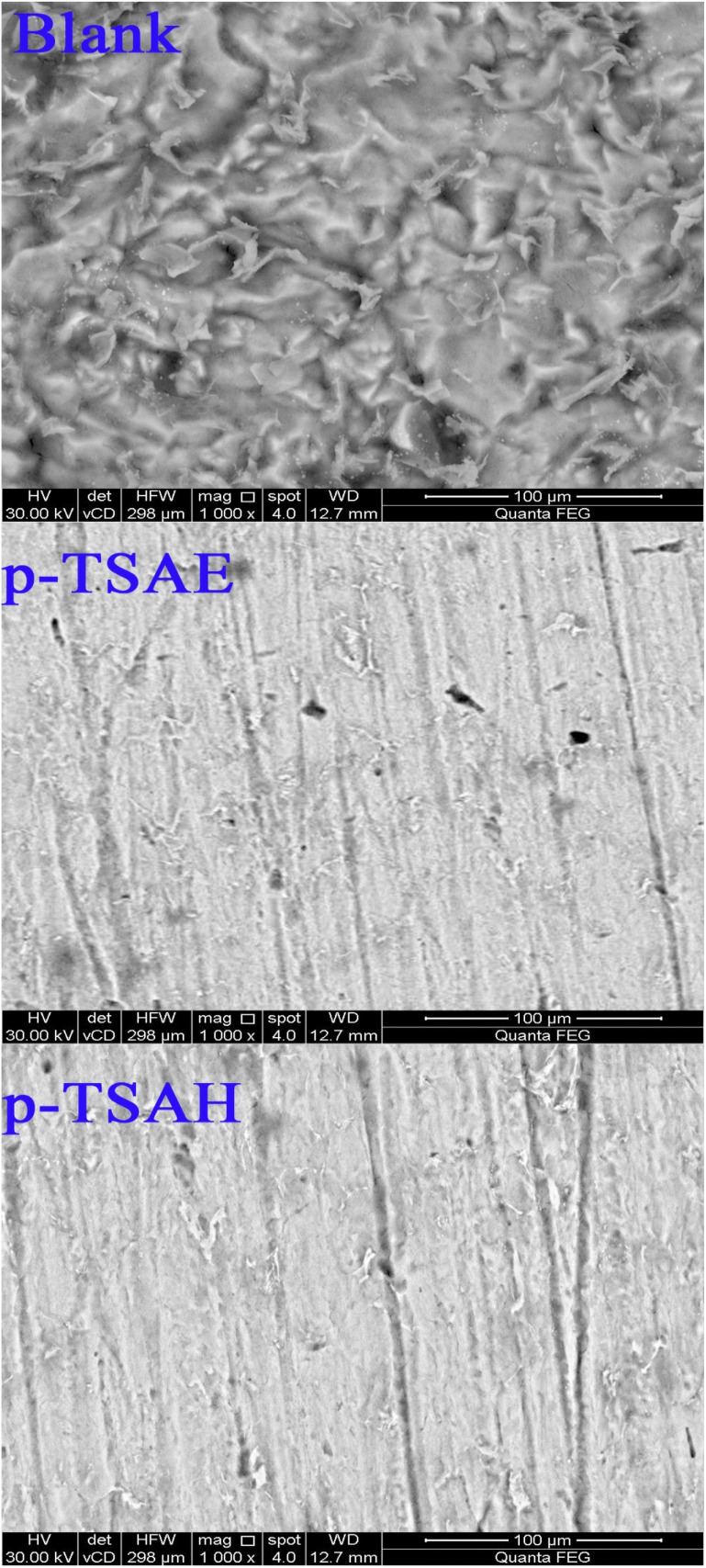
SEM images of the steel surface after 24 hours in the absence and presence of 1.0 × 10^−3^ M of *p*-TSAH and *p*-TSAE compounds at 30 °C.

SEM analysis was performed to confirm the adsorption of *p*-TSAE and *p*-TSAH on the surface of the mild steel specimens. SEM pictures of the morphology of the mild steel samples after 24 hours in the acid solution with and without 1 mM *p*-TSAE and *p*-TSAH are shown in Fig. 10. In the blank sample (mild steel/1.0 M HCl solution), the surface was very rough, badly corroded and destroyed. The addition of *p*-TSAE and *p*-TSAH significantly reduced the extent of the corrosion and the surface became smoother and less damaged. This suggests the formation of an organic protective layer that can effectively resist corrosion.^59^

#### 3.4.2 XPS analysis

**Fig. 11:**
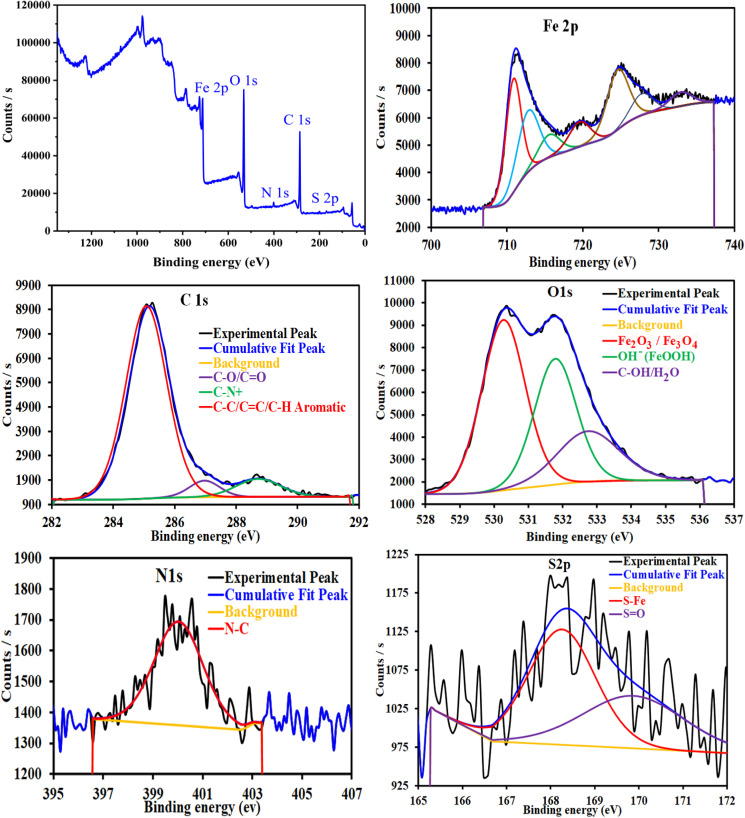
XPS spectra of steel immersed in 1.0 M HCl with the addition of 1.0 × 10^−3^ M of *p*-TSAH.

The XPS technique was used to investigate the formation of an adsorbed organic layer on the steel surface. The analysis was done on mild steel in 1.0 M HCl containing 1.0 × 10^−3^ M of *p*-TSAH after immersion for 24 h and the data is shown in Fig. 11. The surface survey proved the presence of C, N, S and O with atomic percentages of 52.16%, 2.63%, 0.9% and 33.44%, respectively. The deconvoluted spectrum of C1s shows three bands corresponding to C–C/C=C/C–H aromatic (285.18 eV), C–O/C=O (286.97 eV) and C–N, C–N^+^ (288.72 eV). The N1s spectrum shows one peak at 400.03 eV, corresponding to N–C/N–H. The deconvoluted spectrum of O1s shows three bands at 530.28, 531.79 and 532.74 eV, corresponding to several iron oxides such as Fe_2_O_3_/Fe_3_O_4_/FeOOH and organic species such as C–O/C=O/C–OH. The S2p spectrum shows two peaks at 168.26 and 169.87 eV, corresponding to S–Fe/S=O.

### 3.5 Molecular reactivity

#### 3.5.1 Global reactivity

Molecular reactivity is an old expression that was used for many decades in organic synthesis before it was used in corrosion studies. An early use of this expression was to describe the reactivity of an organic molecule as a function of its ability to share electrons from its HOMO to the LUMO of another organic molecule.^60^ The same concept is then applied to the ability of organic inhibitors to donate their electrons to metal d-orbitals, so that corrosion reactions are suppressed.^61^ The reactivity requires a low ionization potential and narrow gap between the HOMO and LUMO so that electrons can be easily reallocated into the metal surface.^62^*p*-TSAH and *p*-TSAE are different only in one functional group, an ester group (with one terminal oxygen) *versus* a hydrazine group (with two terminal nitrogen atoms), which suggests close global reactivities. However, the hydrazine should be slightly more reactive due to the ease of reallocation of the two lone pairs of the two nitrogen atoms. This is shown clearly in the calculated values of the energy gap^63^ as shown in Table 6. The calculated LUMO–HOMO energy difference (Δ*E*) for *p*-TSAH and *p*-TSAE are 5.015 and 5.022 eV, respectively. These values were calculated by subtracting the eigenvalue of the highest occupied molecular orbital (HOMO) from the eigenvalue of the lowest unoccupied molecule orbital (LUMO), both obtained directly from DFT results. IPs (ionization potentials) of 6.228 and 6.440 eV for *p*-TSAH and *p*-TSAE, respectively (Table 6), indicate a slightly greater tendency of *p*-TSAH to donate its HOMO electrons to the metal surface and therefore suppress surface corrosion somewhat more effectively. In addition, both *p*-TSAH and *p*-TSAE show low chemical hardness of 2.511 and 2.507 eV, respectively, which should be reflected in an effortlessness interaction between inhibitor molecules and mild steel. It is worth mentioning that the low hardness is due to the low energy gap – in molecules with filled HOMOs, the electronegativity separates the energy gap into two parts, and their combination gives the chemical hardness.

**Table 6:** Parameters of global reactivity for *p*-TSAH and *p*-TSAE from B3LYP/6-31g(d,p) calculations

Molecular parameters^a^	Mathematical formula	*p*-TSAH	*p*-TSAE
*E*_LUMO_ b	—	−1.213	−1.419
*E*_HOMO_ b	—	−6.228	−6.440
Δ*E*	(*E*_LUMO_ − *E*_HOMO_)	5.015	5.022
Ionization potential (IP)	−*E*_HOMO_	6.228	6.44
Electron affinity (EA)	−*E*_LUMO_	1.213	1.419
Electronegativity (*χ*)	(IP + EA)/2	3.72	3.93
Chemical potential (*μ*)	−*χ*	−3.72	−3.93
Chemical hardness (*η*)	(IP − EA)/2	2.507	2.511
Chemical softness (*σ*)	1/*η*	0.399	0.398
Electrophilicity index (*ω*)	*χ* ^2^/2*η*	2.76	3.075
Δ*N*	(*χ*_metal_ − *χ*_inh_)/2(*η*_metal_ + *η*_inh_)	1.152	1.109

a The units used are eV except for Δ*N* which is a dimensionless quantity.

b EHOMO and ELUMO were taken from DFT computation.

The tendency of electrons to flow between the inhibitor and the metal surface can be estimated using the following equation:^64^7
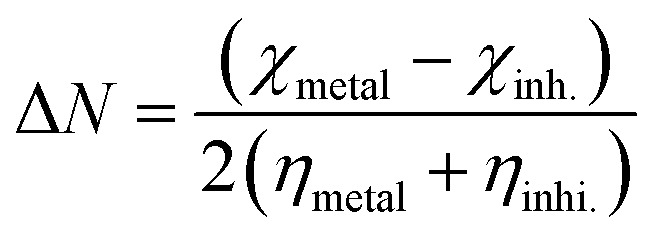
where the absolute value of Δ*N* refers to the direction of the electron flow from an inhibitor to the vacant metal orbitals and thereby is a dimensionless quantity (Table 6). The calculated Δ*N* values for *p*-TSAH and *p*-TSAE are 1.152 and 1.109, respectively, indicating a greater ability of *p*-TSAH to donate electrons to the metal than *p*-TSAE.

**Fig. 12:**
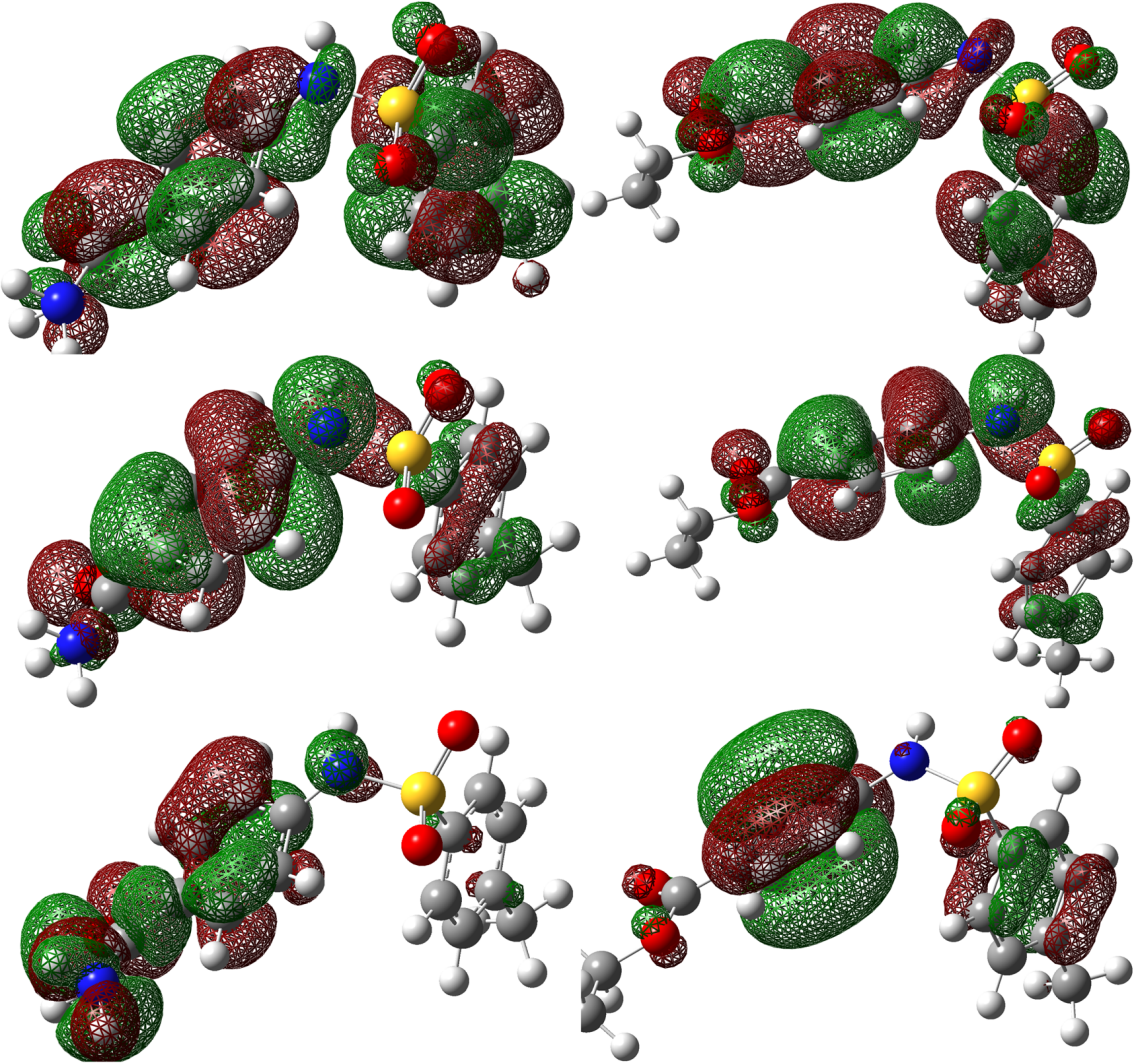
Frontier molecular orbital densities for *p*-TSAH (left) and *p*-TSAE (right); LUMO (upper), HOMO (middle), and HOMO−1 (bottom).

A qualitative description of both the molecular and atomic reactivity can be explored further by investigating both the frontier molecular orbital and natural bond orbital densities. Fig. 12 shows the FMOs for *p*-TSAH and *p*-TSAE, specifically the HOMO and LUMO for each molecule. It shows that the HOMO of the *p*-TSAE molecule is delocalized mainly over the phenyl ring (attached to the ester group), and the nitrogen and oxygen atoms of the ester group, and less so over the other phenyl ring. It is worth noting the missing contribution of the sulfur atom to the HOMO of *p*-TSAE, while having three lone pairs with low electronegativity. This could be explained in that it is surrounded by two oxygen atoms and one nitrogen atom with higher electronegativity (N/O) (3.04/3.44) than sulfur (2.58). A fruitful synthesis of organic inhibitors then demands a delocalized distribution of hetero atoms across the molecule so that they don't cancel the donating ability of each other. The same surface density distribution stands for the HOMO of the *p*-TSAH molecule in Fig. 12, with a lack of electron density over the nitrogen atoms of the hydrazine group, which, instead, contributes to HOMO−1. Overall, the FMO analysis shows the priority of electron donation to the phenyl ring attached to either the ester or hydrazine group with contributions from the oxygen of the carbonyl group and the nitrogen atom at the linkage of the two aromatic rings being similar.

**Fig. 13:**
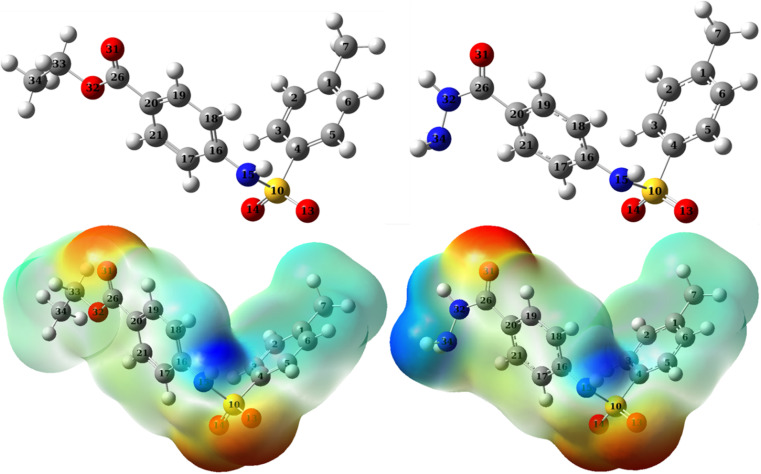
Optimized structures (upper panel) and molecular electrostatic potential (MEP) map (lower panel) of *p*-TSAH (right panel) and *p*-TSAE (left panel).

To evaluate the priority of individual atoms/groups involved in the HOMO of each molecule in sharing their electrons with the metal d-orbitals, the NBOs were analyzed.^65^Tables 7 and 8 show the orbital hybridization schemes for the proposed sites of interactions ordered by the priority of the electron donating ability along with the corresponding eigenvalues in eV. In addition, atom numbering is provided in Fig. 13. The corresponding surface densities are provided in Tables S1 and S2 in the ESI† in the same order. According to Table 7, hybridizations of *p*-TSAH are in the following order: LP (1) C_20_ > LP (2) O_31_ > BD (2) C_17_–C_21_ > BD (2) C_18_–C_19_ > BD (2) C_1_–C_6_ > BD (2) C_2_–C_3_ > BD (2) C_4_–C_5_ > LP (1) N_32_ > LP (2) O_13_ > LP (3) O_13_ > LP (3) O_14_ > LP (2) O_14_ > LP (1) N_15_ > LP (1) N_34_ > BD (2) C_26_–O_31_, which corresponds to the orbitals from HOMO to HOMO−13 (Table S1†). This order corresponds to the abilities of the hybridizations to reallocate their electron density into d-orbitals of the metal; *i.e.* LP (1) C_20_ is at the top of the natural bond orbitals (HOMO), and hence the easiest donation takes place from it, followed by LP (2) O_31_ (HOMO−1), *etc.p*-TSAE shows similar donating behavior according to the sequence: LP (1) (C_20_) > LP (2) (O_31_) > BD (2) C_17_–C_21_ > BD (2) C_18_–C_19_ > BD (2) C_1_–C_6_ > BD (2) C_2_–C_3_ > BD (2) C_4_–C_5_ > LP (3) (O_13_) > LP (3) (O_14_) > LP (2) (O_13_) > LP (2) (O_14_) > LP (1) (N_15_) > LP (2) (O_32_) > BD (2) C_26_–O_31_. These hybridizations correspond to the orbitals from HOMO to HOMO−13 (Table S2†). Therefore, in both molecules most of the donating ability goes to the phenyl ring as well as the carbonyl group attached to it, followed by the other phenyl group and the oxygen atoms of the sulfonyl group. The oxygen and nitrogen atoms of the ester and hydrazine groups, respectively, show the lowest donating ability.

**Table 7:** *p*-TSAH NBOs at expected inhibitor-metal interactions ordered according to their energies (highest to lowest)

Bond	Occupancy	Energy	NBO	s % (atom 1)	p % (atom 1)	s % (atom 2)	p % (atom 2)
LP (1) C_20_	1.057	−0.104	p	0	99.99	—	—
LP (2) O_31_	1.865	−0.237	p	0	99.78	—	—
BD (2) C_17_–C_21_	1.669	−0.246	0.7256 p^1.00^ + 0.6881 p^1.00^	0.02	99.93	0	99.95
BD (2) C_18_–C_19_	1.682	−0.251	0.7329 p^1.00^ + 0.6803 p^1.00^	0.01	99.95	0	99.95
BD (2) C_1_–C_6_	1.627	−0.255	0.6920 p^1.00^ + 0.7219 p^1.00^	0.01	99.95	0	99.96
BD (2) C_2_–C_3_	1.658	−0.259	0.7189 p^1.00^ + 0.6951 p^1.00^	0	99.96	0	99.95
BD (2) C_4_–C_5_	1.689	−0.273	0.7500 p^1.00^ + 0.6614 p^1.00^	0.04	99.95	0	99.95
LP (1) N_32_	1.758	−0.278	sp^21.54^	4.44	95.54	—	—
LP (2) O_13_	1.816	−0.287	p	0.07	99.63	—	—
LP (3) O_13_	1.783	−0.287	p	0.08	99.61	—	—
LP (3) O_14_	1.772	−0.287	p	0.07	99.62	—	—
LP (2) O_14_	1.82	−0.289	p	0.06	99.64	—	—
LP (1) N_15_	1.833	−0.314	sp^8.95^	10.04	89.91	—	—
LP (1) N_34_	1.962	−0.34	sp^3.75^	21.02	78.91	—	—
BD (2) C_26_–O_31_	1.981	−0.405	0.5520 sp^40.39^ + 0.8339 sp^30.29^	2.41	97.4	3.12	96.57

**Table 8:** *p*-TSAE NBOs at expected inhibitor-metal interactions ordered according to their energies (highest to lowest)

Type	Occupancy	Energy	NBO	s % (atom 1)	p % (atom 1)	s % (atom 2)	p % (atom 2)
LP (1) C_20_	1.065	−0.113	p^1.00^	0	100	—	—
LP (2) O_31_	1.849	−0.253	p^0.99^	0.01	99.74	—	—
BD (2) C_17_–C_21_	1.671	−0.255	0.7243 p + 0.6895 p	0	99.94	0	99.95
BD (2) C_18_–C_19_	1.682	−0.258	0.7326 p + 0.6807 p	0	99.95	0	99.95
BD (2) C_1_–C_6_	1.625	−0.259	0.6919 p + 0.7220 p	0.01	99.95	0	99.96
BD (2) C_2_–C_3_	1.657	−0.263	0.7190 p + 0.6950 p	0	99.96	0	99.95
BD (2) C_4_–C_5_	1.688	−0.278	0.7506 p + 0.6608 p	0	99.95	0	99.95
LP (3) O_13_	1.782	−0.291	p^0.99^	0.08	99.61	—	—
LP (3) O_14_	1.771	−0.291	p^0.99^	0.06	99.63	—	—
LP (2) O_13_	1.815	−0.292	p^0.99^	0.07	99.63	—	—
LP (2) O_14_	1.819	−0.293	p^0.99^	0.07	99.63	—	—
LP (1) N_15_	1.829	−0.318	sp^9.36^	9.65	90.31	—	—
LP (2) O_32_	1.796	−0.318	p^1.00^	0	99.91	—	—
BD (2) C_26_–O_31_	1.983	−0.372	0.5512 p + 0.8343 p	0	99.81	0	99.67

#### 3.5.2 Local reactivity

A molecular electrostatic potential (MEP) map divides a molecule into low potential regions and high potential regions corresponding to electron-rich and electron-poor regions, labeled red and blue, respectively (Fig. 13). The electrophile (in this case the metal) tends to interact with the inhibitor through the red sites where it can find electrons. The map shown in Fig. 13 shows that *p*-TSAE has two extreme electron-rich regions (red regions) and one extreme electron-poor region (blue region) while *p*-TSAH has two electron-rich against two electron-poor sites. It is worth noting that the number of electrophile and nucleophile sites doesn't necessarily reflect the overall reactivity of the molecule, however it is an indication of the locations where the metal can interact more effectively. In both *p*-TSAH and *p*-TSAE, the electron-rich sites are those surrounding oxygen atoms while the electron-poor sites are those surrounding nitrogen atoms, presumably due to the withdrawing effect of both oxygen atoms and phenyl rings on the lone pairs of the nitrogen atoms. On the other hand, the green colored sites of the π-system of the phenyl rings indicates moderate electron density. While the NBOs suggested that, on the basis of energy, the phenyl rings respond before the oxygen atoms in inhibitor-metal interactions, the MEP map reveals that oxygen atoms, electron rich sites that come later in the NBO energy scale, provide a greater amount of electron density. Nevertheless, condensed Fukui functions can be used to quantitatively determine the reactivity locations within a molecule on an atom-by-atom basis using the following formulae:^66^

8 *f*^−^_k_ = [*q*_k_(*N*) − *q*_k_(*N* − 1)]

9 *f*^+^_k_ = [*q*_k_(*N* + 1) − *q*_k_(*N*)]

where *q*_k_(*N*), *q*_k_(*N* + 1), and *q*_k_(*N* − 1) are the net charges on the atom k in neutral, cationic and anionic species, respectively. Atoms with higher values of *f*^−^_k_ are subject to electrophilic attack, while atoms with *f*^+^_k_ are subject to nucleophilic attack.^60,67^ The calculated Fukui indices for the *p*-TSAH and *p*-TSAE molecules are listed in Table 9.

**Table 9:** Condensed Fukui functions^a^ for local reactivities in *p*-TSAH and *p*-TSAE molecules

*p*-TSAH			*p*-TSAE		
Atoms	*f* ^+^	*f* ^−^	Atoms	*f* ^+^	*f* ^−^
C_1_	0.035	0.057	S_10_	0.019	0.123
O_31_	0.076	0.056	O_31_	0.049	0.057
S_10_	0.022	0.047	C_26_	0.013	0.055
O_13_	0.04	0.043	C_1_	0.039	0.051
C_26_	0.019	0.043	C_20_	0.079	0.045
C_20_	0.075	0.042	C_16_	0.047	0.042
C_4_	0.012	0.037	C_21_	0.036	0.034
C_5_	0.021	0.037	O_13_	0.043	0.034
C_6_	0.022	0.036	C_19_	0.038	0.033
C_16_	0.05	0.036	C_6_	0.024	0.029
C_3_	0.006	0.035	C_18_	0.047	0.028
C_21_	0.034	0.034	C_4_	0.015	0.027
O_14_	0.028	0.033	C_5_	0.023	0.024
C_19_	0.038	0.033	O_14_	0.032	0.024
C_2_	0.024	0.028	C_17_	0.052	0.024
C_18_	0.044	0.027	C_3_	0.008	0.022
C_17_	0.051	0.025	C_2_	0.028	0.021
N_32_	0.021	0.023	O_32_	0.014	0.014
N_15_	0.083	0.018	C_7_	0.013	0.007
C_7_	0.012	0.017	C_33_	0.008	0.001
N_34_	0.013	0.008	C_34_	0.006	−0.003
—	—	—	N_15_	0.095	−0.022

a Calculated using Hirshfeld charges at B3LYP/6-31G(d,p).

The electrophilic Fukui indices *f*^−^ show that when a metal surface approaches *p*-TSAE, the S_10_ atom (*f*^−^ = 0.123) has the largest electronic density contribution to the d-orbitals of the metal, however, on the basis of energy, because S_10_ orbitals come late within the NBOs, it should have a low probability of approaching the metal surface. On the other hand, phenyl rings show high electrophilic contributions to the metal surface, in agreement with the order of the NBOs, which confirms their ability to adhere to the metallic surface. The atoms of the *p*-TSAH molecule have close electrophilic Fukui indices to those of the atoms of *p*-TSAE molecule, however the nitrogen atoms of the hydrazine group of the *p*-TSAH molecule have the lowest electrophilic indices (N_32_ and N_34_ are 0.023 and 0.008, respectively) which confirms the electrochemical results in which *p*-TSAH has better corrosion inhibition than *p*-TSAE.

### 3.6 Monte Carlo simulations

**Fig. 14:**
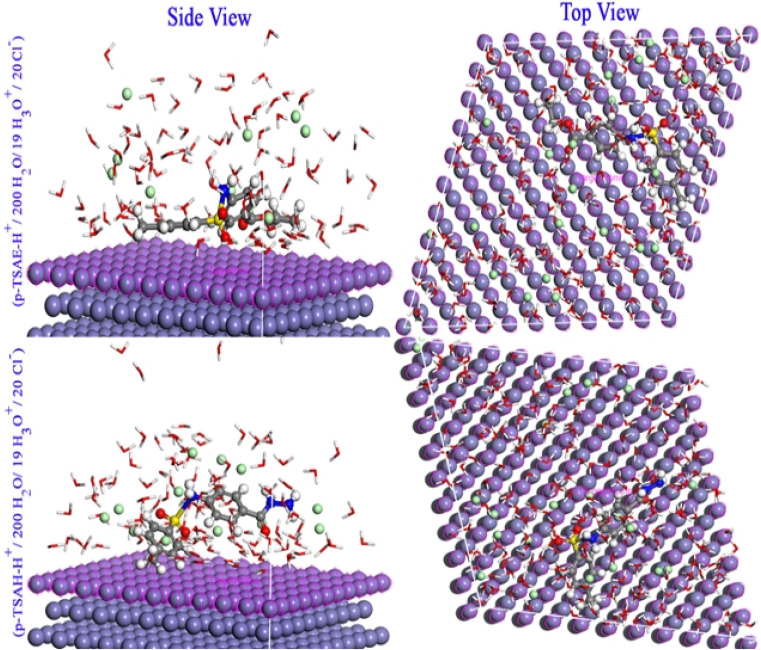
Monte Carlo simulations for the most favorable modes of adsorption obtained for *p*-TSAE-H^+^ and *p*-TSAH-H^+^ on the Fe (1 1 0) surface, side and top view.

MC simulations were used to identify the inhibitor molecule (*p*-TSAH and *p*-TSAE) attractions to the Fe surface as well as to offer a clear understanding of the adsorption mechanism. As a result, Fig. 14 shows that the most stable adsorption configuration for the protonated inhibitors is approximately flat. This flat and parallel orientation achieves the highest proper adsorption formations for *p*-TSAH and *p*-TSAE molecules on the Fe interface in the acidic solution, implying an enhancement in the adsorption and maximum surface coverage.^68^ As a result, we may conclude that *p*-TSAH and *p*-TSAE molecules prefer to interact with the Fe surface even after protonation through several functional groups, heteroatoms, and π bonds. Table 10 contains the adsorption and binding energy parameters. The results demonstrate that the examined *p*-TSAH and *p*-TSAE molecules exhibit substantial negative adsorption energy, which supports the enhanced and spontaneous inhibitory effect of these compounds on the mild steel surface. These findings suggest that the adsorbed H_2_O and HCl molecules on the Fe (110) surface can be gradually substituted by inhibitor molecules, resulting in the creation of a protective film between the metallic surface and the aqueous corrosive solution.^69^

**Table 10:** The outputs and descriptors calculated by the Monte Carlo simulations for the adsorption of *p*-TSAH and *p*-TSAE on Fe (110) (in kcal mol^−1^)

Phase	Inhibitor	Total energy (kcal mol^−1^)	Adsorption energy (kcal mol^−1^)	Rigid adsorption energy (kcal mol^−1^)	Deformation energy (kcal mol^−1^)	(d*E*_ads_/dNi) (kcal mol^−1^)	Binding energy (kcal mol^−1^)	IE^a^ (%)
Gas phase	*p*-TSAE	−111.363	−550.819	−161.848	−388.971	−550.819	550.819	92.51
	*p*-TSAH	−173.753	−622.299	−177.272	−445.026	−622.299	622.299	94.32
	*p*-TSAE-H^+^	−145.089	−734.915	−175.178	−559.736	−734.915	734.915	92.51
	*p*-TSAH-H^+^	−205.944	−752.517	−190.172	−562.344	−752.517	752.517	94.32
Aqueous phase	*p*-TSAE	−5966.133	−6565.058	−6156.983	−408.075	−478.562	478.562	92.51
	*p*-TSAH	−5975.518	−6583.533	−6113.947	−469.586	−517.565	517.565	94.32
	*p*-TSAE-H^+^	−5939.723	−6695.038	−6105.430	−589.608	−668.719	668.719	92.51
	*p*-TSAH-H^+^	−6094.487	−6806.550	−6225.349	−581.200	−866.154	866.154	94.32

a Inhibition efficiency values obtained from EFM measurements.

### 3.7 The inhibition mechanism

**Fig. 15:**
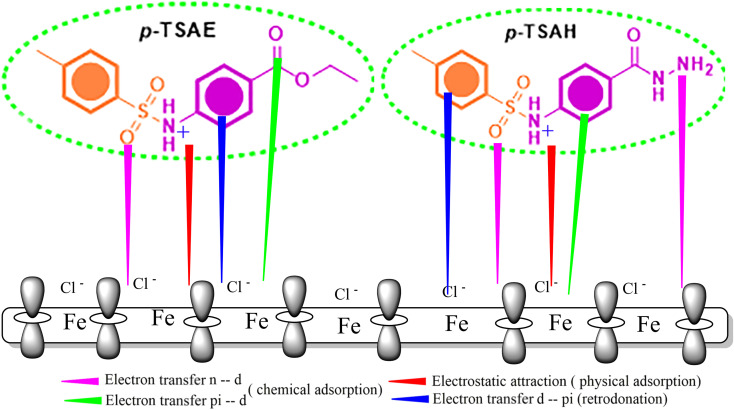
Pictorial representation of the adsorption of *p*-TSAE and *p*-TSAH on the steel surface in 1.0 M HCl.

The adsorption process on the metal interface is the key factor in determining the inhibition capacity of the organic molecules. This adsorption process includes the formation of a protective barrier layer over the metal surface, which shields it from aggressive components.^70^Fig. 15 shows the adsorption modes of *p*-TSAH and *p*-TSAE in order to inhibit the mild steel corrosion in a hydrochloric acid solution. As indicated from the Langmuir isotherm model and the values of Δ*G*, our investigated inhibitors are mixed type between the physical and chemical adsorption modes, and the chemical mode predominates. This is in addition to the parallel adsorption configuration through multiple active sites deduced from the computational calculations. As a result, the electrons of the aromatic moieties, lone pairs of electrons on the hetero atoms (S, N and O) and π electrons (C=O and S=O), can exchange their electrons with the vacant “d” orbitals of Fe as chemical adsorption modes to limit further corrosion of the metal exposed to the corrosive solution.^71^ In the hydrochloric acid solution, the *p*-TSAH and *p*-TSAE molecules can be easily protonated. Thus, the electrostatic interaction between the positively charged protonated inhibitor species and the negatively charged Cl^−^ ions (originally bonded to the steel surface) generates the physisorption mode. Furthermore, the extra electrons on the steel surface can be transferred back to the empty π* regions of the inhibitor molecules (retro-donation).^72^

## 4. Conclusions

(1) The facile synthesis of two corrosion inhibitors has been achieved in a large, scalable approach based on the Hinsberg reaction, forming commercially available chemicals that can be applied in industrial applications.

(2) The investigated synthesized compounds *p*-TSAH and *p*-TSAE were highly efficient inhibitors of corrosion, while *p*-TSAH had a better inhibition efficiency than *p*-TSAE in an acid medium.

(3) The adsorption of *p*-TSAH and *p*-TSAE on steel was tested against several isotherms and found to be in line with the Langmuir model.

(4) The adsorption of the compounds is of mixed type and controlled by the charge transfer mechanism.

(5) The design of organic inhibitors requires a delocalized distribution of hetero atoms across the molecule to achieve the optimal donating ability of these atoms.

(6) The active adsorption regions were confirmed using NBO and MC analysis.

## Conflicts of interest

The authors declare that they have no known competing financial interests or personal relationships that could have appeared to influence the work reported in this paper.

## Supplementary Material

RA-013-D2RA05939H-s001
