# Supplementary material for: Ethyl ester/acyl hydrazide-based aromatic sulfonamides: facile synthesis, structural characterization, electrochemical measurements and theoretical studies as effective corrosion inhibitors for mild steel in 1.0 M HCl
Source: RSC Adv. 2022 Dec 20;13(1):186–211. doi: 10.1039/d2ra05939h (PMC9764999; doi:10.1039/d2ra05939h)

Electronic Supplementary Material (ESI) for RSC Advances.
This journal is © The Royal Society of Chemistry 2022

## Supporting Information for

# An Ethyl Ester/Acyl Hydrazide-Based Aromatic Sulfonamide: Facile Synthesis, Structural Characterization, Electrochemical Measurements and Theoretical Studies as Effective Corrosion Inhibitors for Mild Steel in 1.0 M HCl

Mahmoud A. Bedair[a,b], Ahmed M. Abuelela[a,*], Medhat Owda[a], Essam M. Eliwa[a]

[a]*Department of Chemistry, Faculty of Science (Men's Campus), Al-Azhar University, Nasr City 11884, Cairo, Egypt*
[b]*College of science and arts, University of Bisha, Al-Namas 61977, P.O. Box 101, Saudi Arabia.*

**Table S1** Calculated NBOs densities of *p*-TSAH at expected inhibitor-metal interactions.

| | |
|---|---|
| LP(1)$C_{20}$ | LP(2)$O_{31}$ |
| 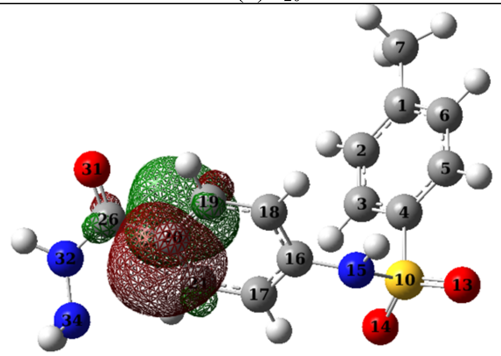 | 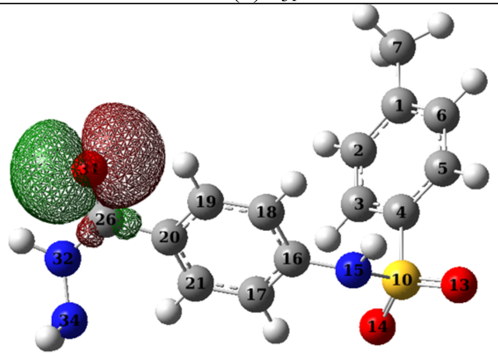 |
| BD(2)$C_{17}$-$C_{21}$ | BD(2)$C_{18}$-$C_{19}$ |
| 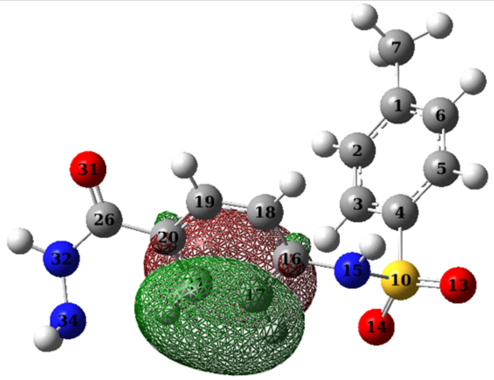 | 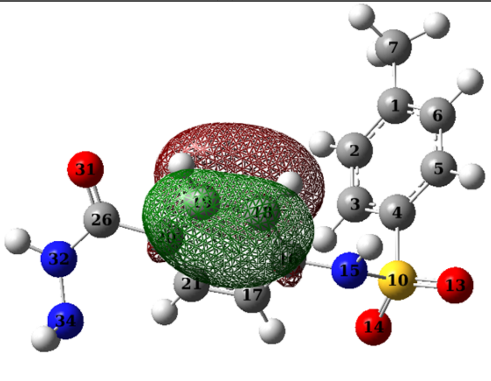 |
| BD(2)$C_1$-$C_6$ | BD(2)$C_2$-$C_3$ |
| 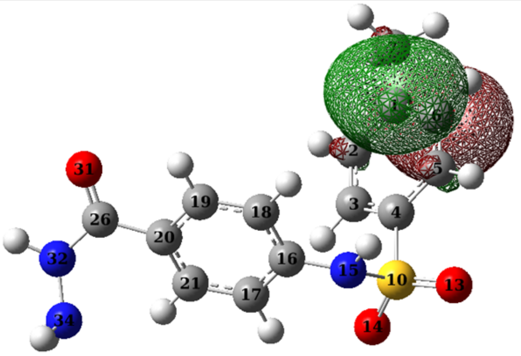 | 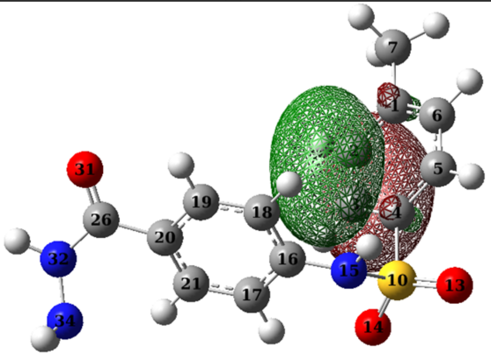 |
| BD(2)$C_4$-$C_5$ | LP(1)$N_{32}$ |

| | |
|---|---|
| 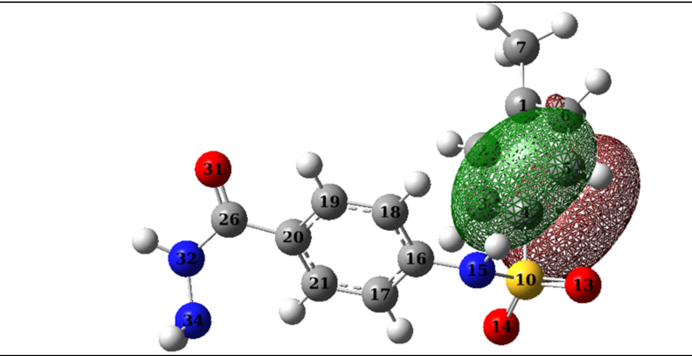 | 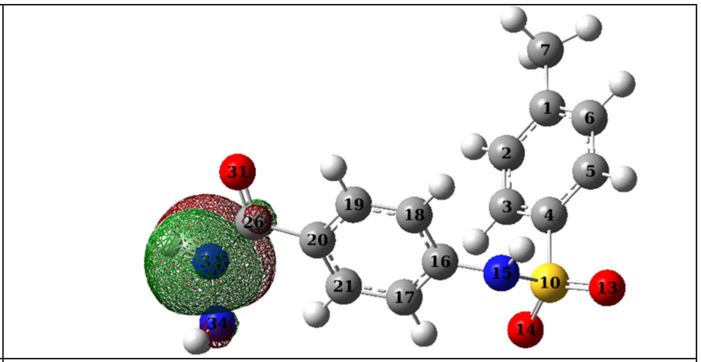 |
| LP(2)$O_{13}$ | LP(3)$O_{13}$ |
| 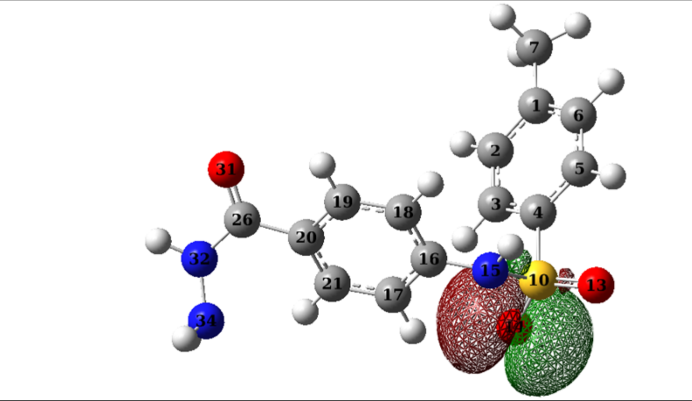 | 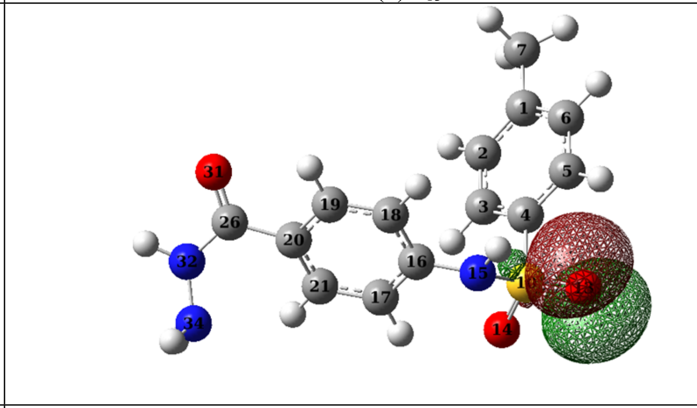 |
| LP(3)$O_{14}$ | LP(2)$O_{14}$ |
| 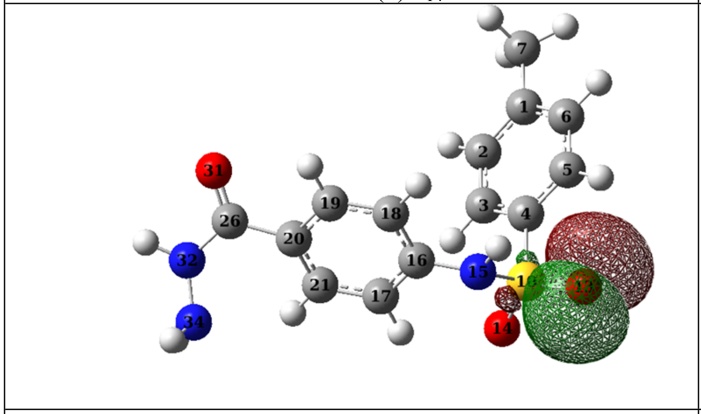 | 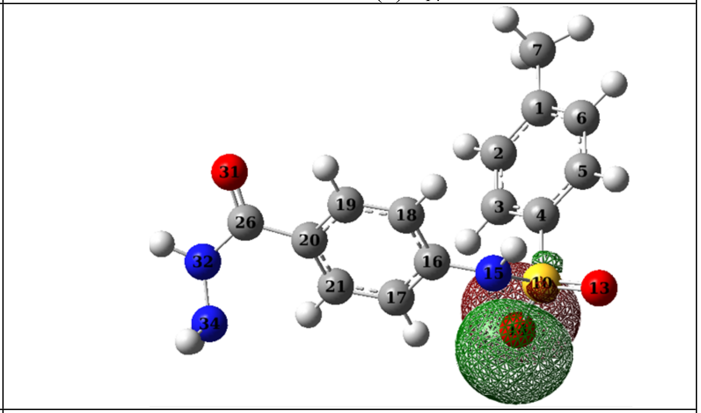 |
| LP(1)$N_{15}$ | LP(1)$N_{34}$ |
| 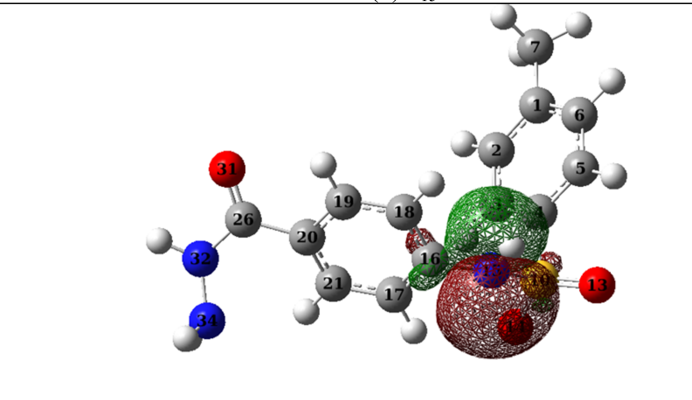 | 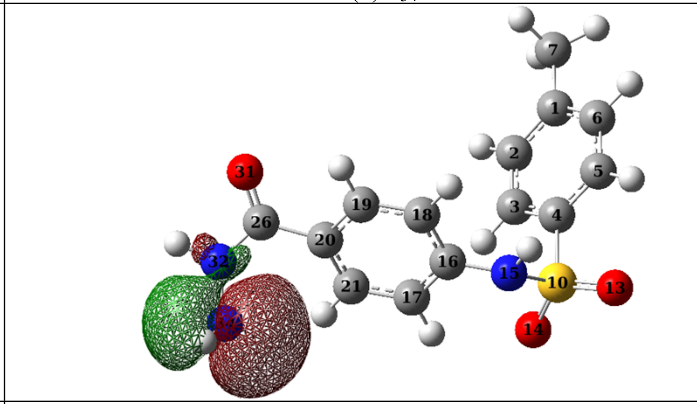 |
| BD(2)$C_{26}$-$O_{31}$ | |

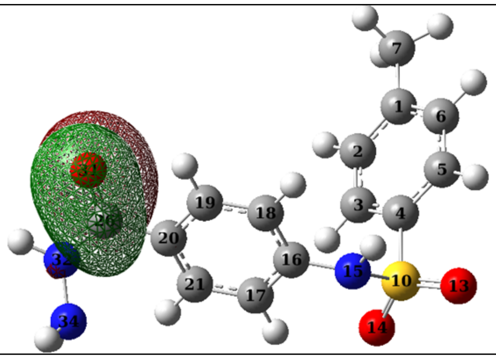
7
1
6
2
5
19
18
3
4
20
16
15
10
13
21
17
14
32
34

**Table S2** Calculated NBOs densities of *p*-TSAE at expected inhibitor-metal interactions.

| | |
|---|---|
| $LP(1)C_{20}$ | $LP(2)O_{31}$ |
| 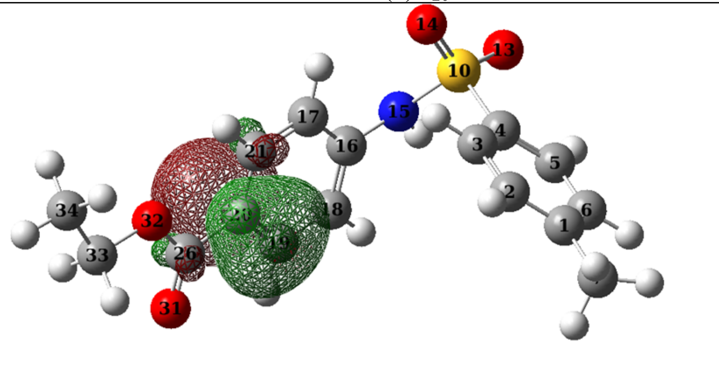 | 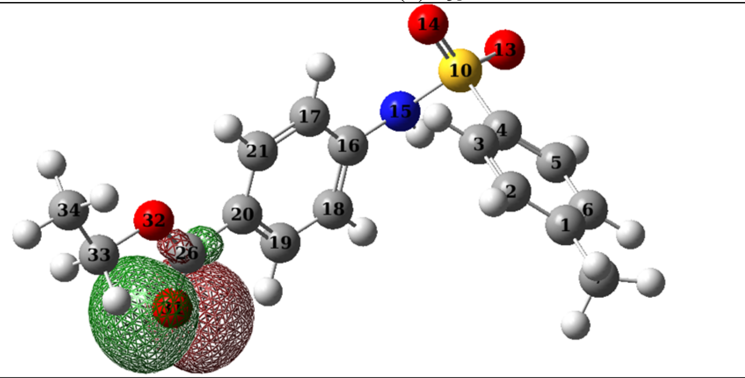 |
| $BD(2)C_{17}-C_{21}$ | $BD(2)C_{18}-C_{19}$ |
| 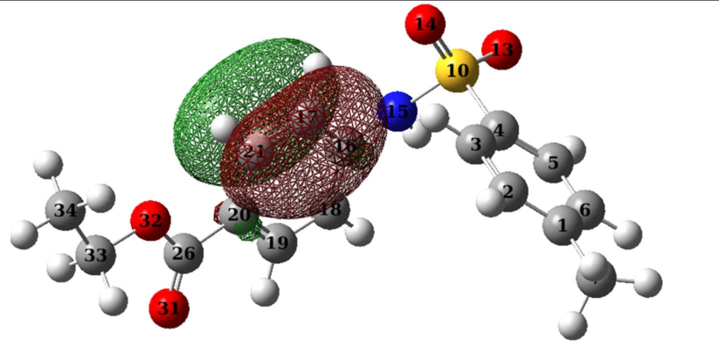 | 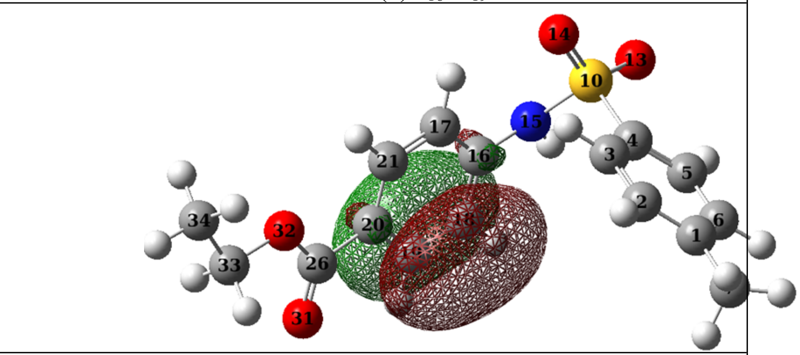 |
| $BD(2)C_{1}-C_{6}$ | $BD(2)C_{2}-C_{3}$ |
| 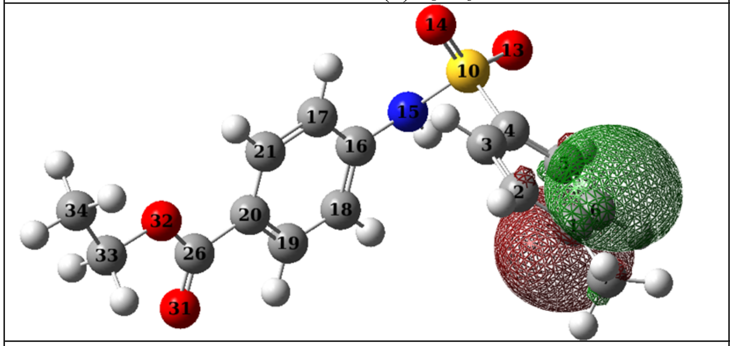 | 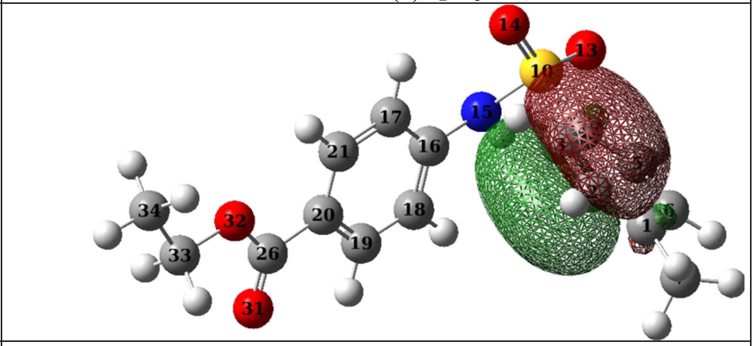 |
| $BD(2)C_{4}-C_{5}$ | $LP(3)O_{13}$ |
| 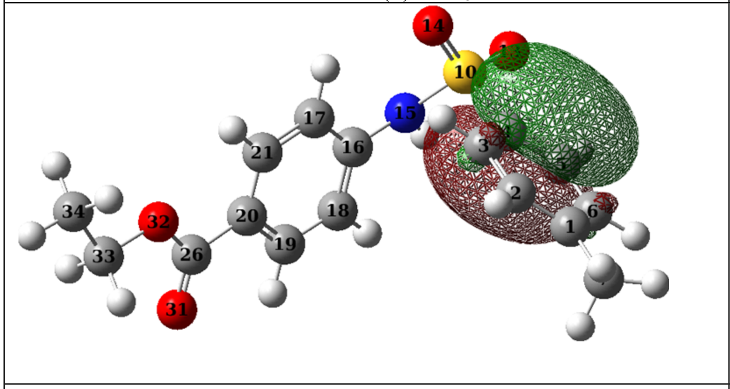 | 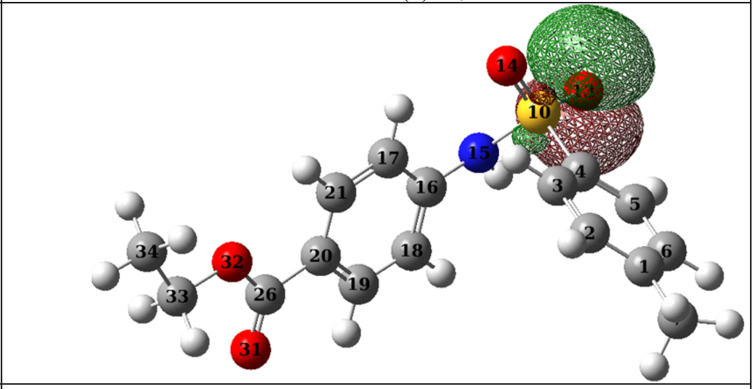 |
| $LP(3)O_{14}$ | $LP(2)O_{13}$ |

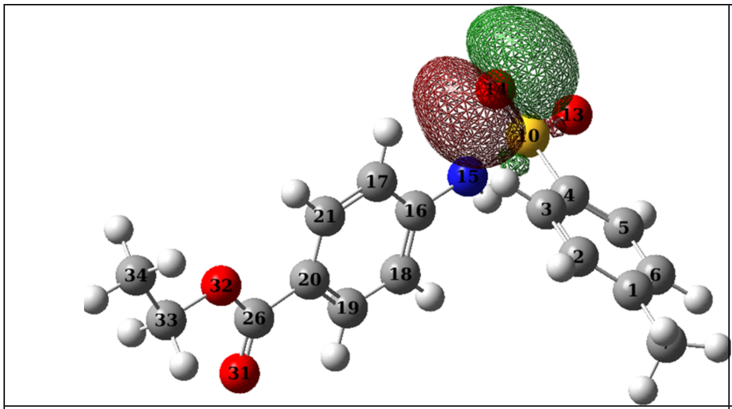


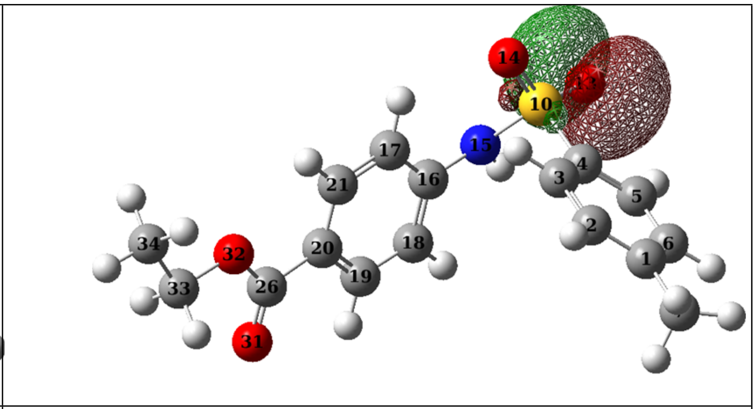


| LP(2)O$_{14}$ | LP(1)N$_{15}$ |
|---|---|

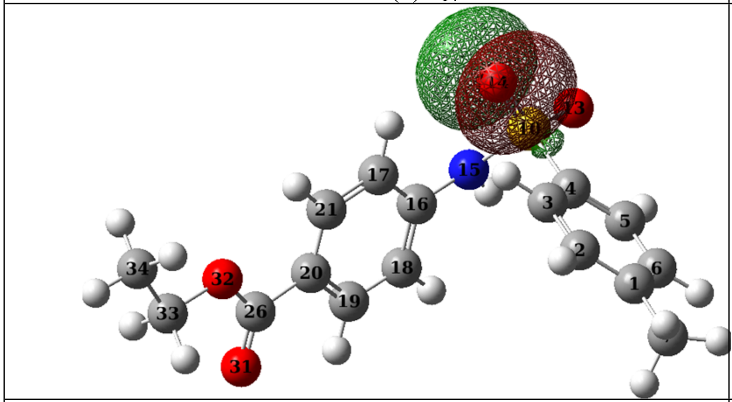


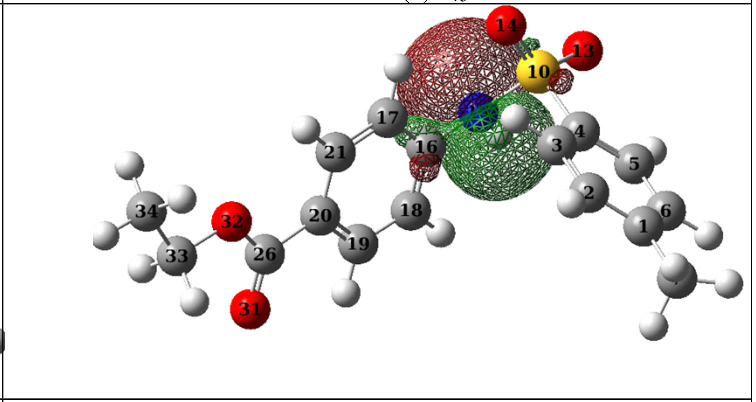


| LP(2)O$_{32}$ | BD(2)C$_{26}$-O$_{31}$ |
|---|---|

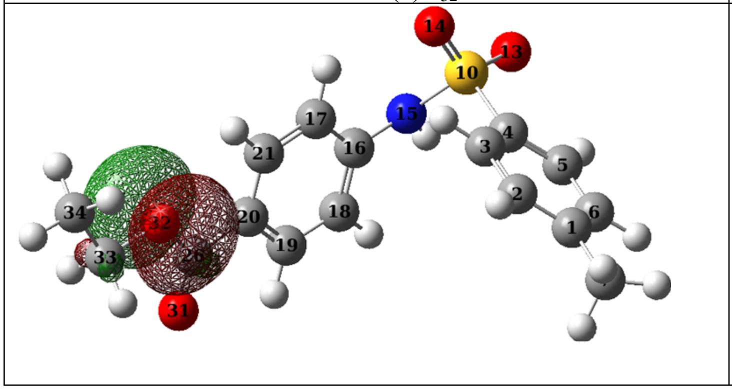


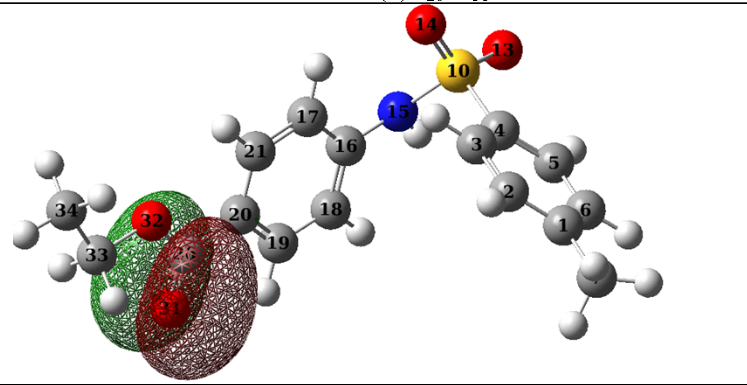

Supplement: RA-013-D2RA05939H-s001 [file RA-013-D2RA05939H-s001.pdf]
